# Viral growth factor- and STAT3 signaling-dependent elevation of the TCA cycle intermediate levels during vaccinia virus infection

**DOI:** 10.1371/journal.ppat.1009303

**Published:** 2021-02-02

**Authors:** Anil Pant, Lara Dsouza, Shuai Cao, Chen Peng, Zhilong Yang

**Affiliations:** Division of Biology, Kansas State University, Manhattan, Kansas, United States of America; University of Washington, UNITED STATES

## Abstract

Metabolism is a crucial frontier of host-virus interaction as viruses rely on their host cells to provide nutrients and energy for propagation. Vaccinia virus (VACV) is the prototype poxvirus. It makes intensive demands for energy and macromolecules in order to build hundreds and thousands of viral particles in a single cell within hours of infection. Our comprehensive metabolic profiling reveals profound reprogramming of cellular metabolism by VACV infection, including increased levels of the intermediates of the tri-carboxylic acid (TCA) cycle independent of glutaminolysis. By investigating the level of citrate, the first metabolite of the TCA cycle, we demonstrate that the elevation of citrate depends on VACV-encoded viral growth factor (VGF), a viral homolog of cellular epidermal growth factor. Further, the upregulation of citrate is dependent on STAT3 signaling, which is activated non-canonically at the serine727 upon VACV infection. The STAT3 activation is dependent on VGF, and VGF-dependent EGFR and MAPK signaling. Together, our study reveals a novel mechanism by which VACV manipulates cellular metabolism through a specific viral factor and by selectively activating a series of cellular signaling pathways.

## Author summary

Vaccinia virus (VACV) is a large DNA virus with an acute and increasing demand for energy and macromolecules to build hundreds and thousands of viral particles in a single cell within hours of infection. The demand postulates reprogramming of the TCA cycle, as it is the central metabolic hub of a cell that generates metabolites for energy production and macromolecule synthesis. We show that VACV infection reprograms cellular metabolism globally, elevating the TCA cycle intermediate levels and modulating related cell metabolism. The elevation of the TCA cycle intermediates depends on the virus-encoded growth factor that stimulates non-canonical STAT3 signaling during VACV infection. Our results provide the metabolic foundation of viral growth factor to boost VACV infection.

## Introduction

Viruses do not have metabolism and rely on their host cells for energy and molecular precursors to replicate. Different viral infections often have different metabolic needs from their host cells. Hence, many viruses have developed strategies to rewire cellular metabolism, and often this ability shapes the outcome of virus replication [1–3]. While metabolism is arguably a hot frontier of virus-host interaction, the molecular mechanisms underlying virus-induced metabolic reprogramming are mostly unknown. Identifying the mechanisms by which a virus usurps host cell metabolism will facilitate understanding viral infection and uncover fundamental mechanisms of metabolic regulation.

Vaccinia virus (VACV), the prototypic member of the *poxviridae* family, is a large, enveloped virus with a double-stranded DNA genome that encodes over 200 genes [4]. It had been used as the vaccine to eradicate smallpox, one of the deadliest diseases in human history [5]. Poxviruses continue to cause significant morbidity and mortality in humans and animals. There are also concerns about unregistered smallpox virus stocks that could be used for bioterrorism [6–8]. In addition, the study of VACV is of great importance because of promising development in its use to treat cancers [9], to produce recombinant proteins [10], and to develop vaccines against other infectious diseases [11]. Recent evidence suggests that VACV is an outstanding model to study how a virus reprograms cellular metabolism. VACV rewires host metabolism such that it upregulates glutamine metabolism [12,13]. It also depends on *de novo* fatty acid synthesis to generate an energy-favorable environment [14], suggesting the virus may need to modulate fatty acid synthesis. We have shown that VACV selectively upregulates the translation efficiency of oxidative phosphorylation (OXPHOS) mRNAs, indicating the requirement of increased and continuous supply of energy during virus replication [15].

Interestingly, while these metabolic alterations by VACV could converge to the tricarboxylic acid cycle (TCA cycle), little is known about how VACV infection impacts the TCA cycle. Citrate, the first intermediate of the TCA cycle and the primary source of cytosolic fatty acid synthesis, stands at the crossroads of these two critical processes in cellular metabolism [16]. Not surprisingly, citrate metabolism contributes to the growth and proliferation of organisms ranging from algae, fungi, bacteria, plants and worms to mammalian cells [17–22]. Given the vital role of this metabolite, it is conceivable that its biosynthesis and breakdown would be affected by many viruses. However, very little is known about how a viral infection may affect this key metabolite of cell metabolism.

VACV encodes two copies of viral growth factor (VGF) gene, C11R, in the inverted terminal repetition (ITR) of its genome. VGF is a viral polypeptide with homology to cellular epidermal growth factor (EGF) and transforming growth factor [23–26]. It is the most highly expressed gene among the 118 early genes during VACV infection [27,28]. This secreted protein induces proliferative effects on VACV-infected cells [29–31], and facilitates cell motility and virus spread [32]. VGF brings about these effects by binding to the EGF receptor (EGFR) to stimulate the mitogen associated protein kinase (MAPK) signaling [33]. The majority of cells in an animal are in resting status and it was shown that VACV with the VGF gene deleted has a reduced replication in resting cells [34]. VGF gene deleted VACV is significantly less virulent in mice [34,35]. The proliferative response generation needs heightened energy and macromolecule metabolism, which depends on the TCA cycle [36,37]. These arguments suggest that VACV VGF could be a key regulator to reprogram host metabolism during VACV infection.

In this study, we report that VACV infection elevates the levels of citrate and other intermediates of the TCA cycle and modulates metabolites closely related to the TCA cycle. We demonstrate that the increased citrate level upon VACV infection depends on VGF expression and cellular EGFR and MAPK signaling. We show that VACV infection induces selective upregulation of non-canonical signal transducer and activator of transcription 3 (STAT3) phosphorylation at the serine727 (S727) via VGF, EGFR, and MAPK signaling. Remarkably, the STAT3 signaling is also required for citrate level elevation during VACV infection. We further demonstrate that the elevation of TCA cycle intermediate levels and VGF-mediated upregulation of non-canonical STAT3 phosphorylation could be independent of glutamine metabolism. These findings identify a novel function of VGF that is needed to reprogram cellular metabolism through a molecular mechanism involving non-canonical STAT3 activation. VGF could be of great utility in understanding how growth factors modulate cellular metabolism and cellular metabolic engineering.

## Results

### VACV infection induces profound reprogramming of cellular metabolism globally under glutamine depleted conditions

**Fig 1:**
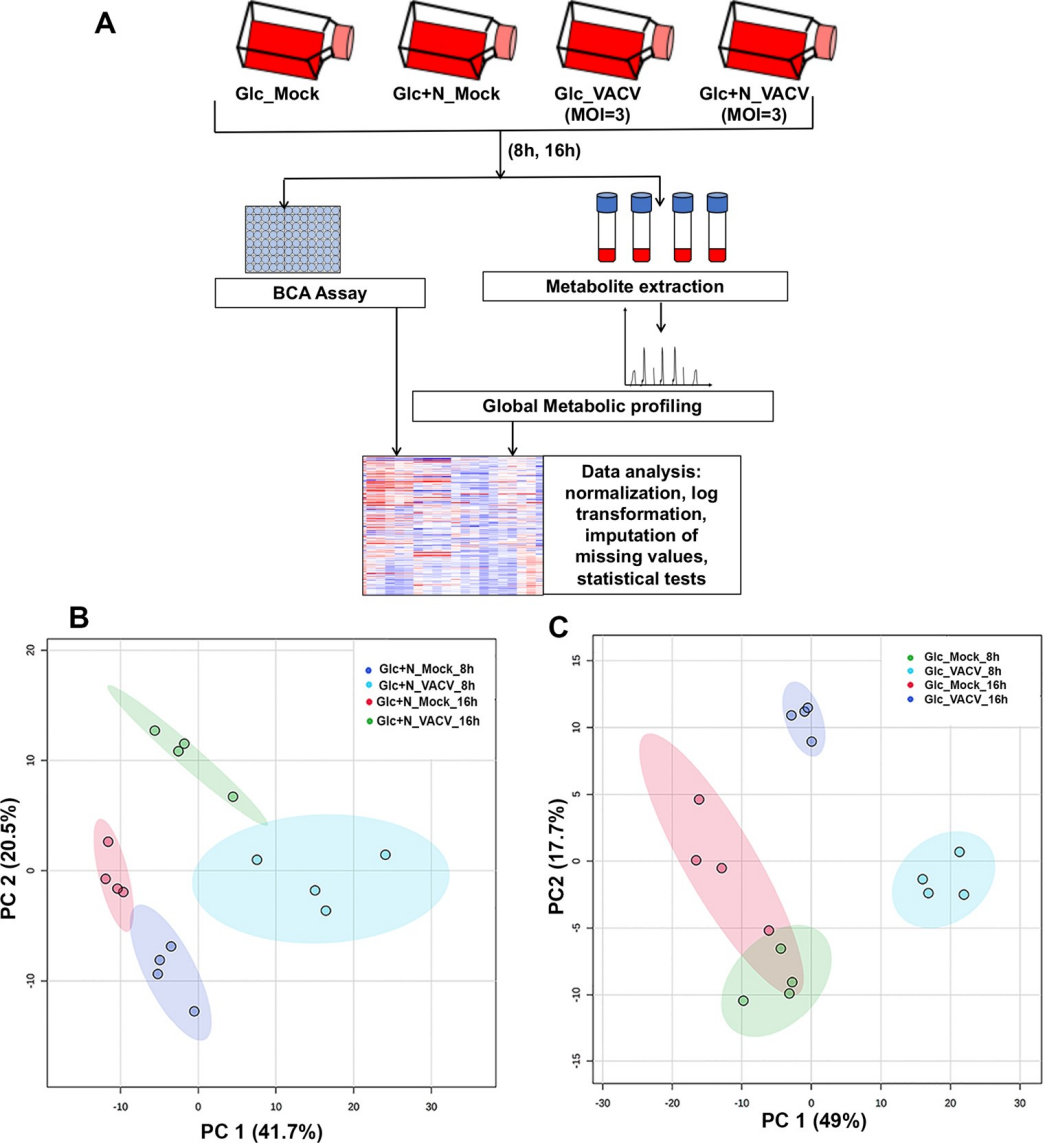
VACV infection reprograms cellular metabolism profoundly and globally under the glutamine-depletion conditions. **(A)** Experimental design of global metabolic profiling. Four biological replicates of HFFs per treatment were either mock-infected or infected with VACV at an MOI of 3 for either 8 or 16 hours in medium with glucose (Glc) or glucose plus asparagine (Glc+N). Metabolites were extracted, and their levels were measured. **(B & C)** Principal component analysis (PCA) showing a clear separation between VACV-infected and uninfected HFFs in glucose plus asparagine medium **(B)** and in HFFs in glucose only medium **(C).** Each small circle indicates one sample. The shaded region indicates the 95% confidence interval. PC1 represents the effect of VACV infection and PC2 represents the effect of time.

VACV replication is substantially reduced in cells cultured in glucose-containing, but glutamine-depleted medium [12,14]. We have previously shown that VACV replication is not affected in medium containing glucose and asparagine under glutamine-depleted condition [38]. As previous studies have shown, VACV upregulates glutaminolysis [12,13], our finding that asparagine can fully rescue VACV replication from glutamine-depletion provides a valuable system to study how VACV modulates cellular metabolism in a glutamine-independent manner. Metabolic profiling during VACV infection in the presence of both glucose and glutamine had been carried out by Fontaine et al. previously [12]. To obtain a global view of the host cell metabolic changes upon VACV infection under the glutamine-depletion condition, we performed metabolic profiling to compare the levels of metabolites in VACV-infected and mock-infected human foreskin fibroblasts (HFFs) cultured in medium containing glucose plus asparagine at 8 and 16 hours post-infection (hpi) (**Fig 1A**). At 8 hpi, the virus is actively replicating, while the virus has completed most of the replication cycle at 16 hpi. We chose the HFFs because they are primary cells, and the metabolism in these cells is not already dysregulated as it is in transformed cancer cells.

In media with glucose plus asparagine, our metabolic profiling detected 173 and 190 metabolites significantly altered by VACV infection, with a general increase at 8 hpi (109 up, 64 down) and decrease at 16 hpi (51 up, 139 down), respectively (**S1 File**). Significant changes in metabolites were prominent in the categories of TCA cycle, amino acids, and carnitylated fatty acids that are used for β-oxidation (**S1 Fig, S2 File**). The substantial changes in cellular metabolism upon VACV infection were clearly revealed by a Principal Component Analysis (PCA), a statistical procedure to summarize the information content in large datasets. (**Fig 1B**).

VACV replication is classified into three stages; early, intermediate, and late, as a cascade based on the timing of its gene expression [4]. Our previous study indicated that VACV replication was not affected at the early gene expression stage but was blocked at intermediate and late replication stages in the absence of glutamine and asparagine in the glucose-containing medium [38]. We also carried out metabolic profiling of VACV-infected and mock-infected HFFs cultured in medium containing glucose without glutamine and asparagine (**Fig 1A**). In glucose only medium, we found 220 and 145 metabolites significantly altered by VACV infection at 8 hpi (156 up, 64 down) and 16 hpi (95 up, 50 down), respectively (**S1 File**). Interestingly, while we observed a similar global metabolic reprogramming pattern as in the glucose plus asparagine medium at 8 hpi, more metabolites were still up in glucose only medium compared to glucose plus asparagine medium at 16 hpi (**Fig 1C, S1 Fig, S2 File**), likely because more nutrients are used in the asparagine containing medium at the later stage of replication, in which VACV replication rate is much higher [38]. These results suggest that the metabolic reprogramming by VACV starts at the early stages of replication. At the later stage, the metabolites are likely consumed to support virus replication.

### VACV infection elevates TCA cycle intermediate levels, including citrate

**Fig 2:**
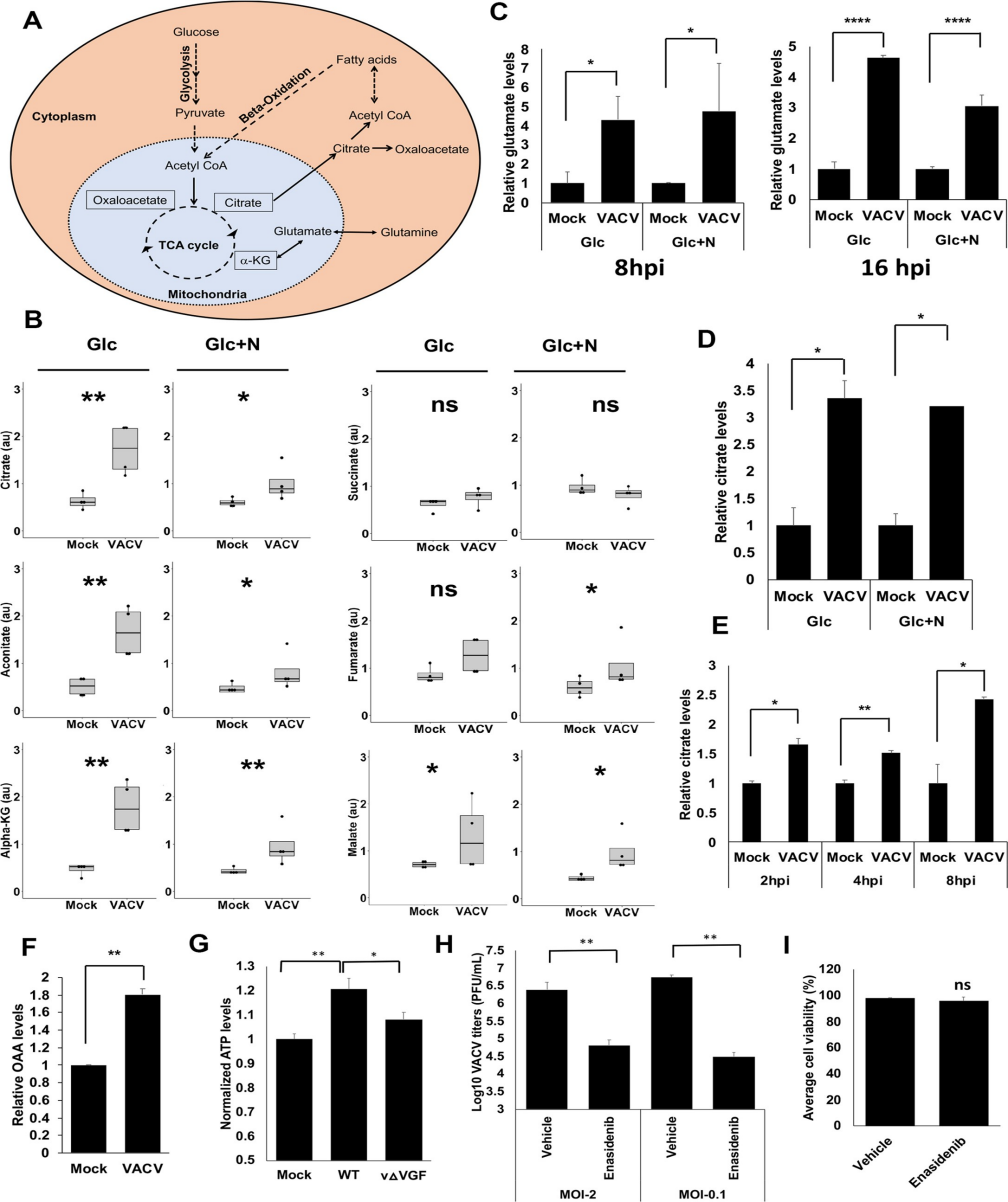
VACV infection elevates the levels of TCA cycle intermediates, including citrate. **(A)** A simplified overview of the TCA cycle and citrate metabolism. The pyruvate generated from glycolysis can be converted into Acetyl-CoA that reacts with OAA to form citrate in the mitochondria of a cell. The citrate can then be transported out of the mitochondria where it gets converted to Acetyl-CoA and OAA. The cytosolic Acetyl-CoA can act as a precursor for fatty acid biosynthesis. The fatty acids undergo β-oxidation in the mitochondria to convert into Acetyl-CoA to feed the TCA cycle. Glutamine can also feed in the TCA cycle to increase the citrate level by converting it to α-KG. **(B)** VACV infection increases the levels of most of the TCA cycle intermediates in the absence of exogenous glutamine. The levels of TCA cycle intermediates at 8 hpi in the metabolic profiling of Fig 1A were shown. **(C)** VACV infection increases the level of glutamate. The level of glutamate in HFFs in the global metabolic profiling of Fig 1A were shown. **(D)** VACV infection increases the citrate level in HFFs cultured in medium without exogenous glutamine. HFFs infected with indicated viruses at MOI of 5 in media with glucose only (Glc) or glucose plus asparagine (Glc+N). Citrate level was measured at 8 hpi using a citrate assay kit. **(E)** VACV infection increases the citrate level in HFFs cultured in medium with glutamine. HFFs infected with WT VACV at an MOI of 5 in medium with glucose plus glutamine and the citrate level was measured at indicated time points using a citrate assay kit. **(F)** VACV infection increases the levels of OAA. HFFs infected with WT VACV at MOI of 5 in HFFs cultured in medium with glucose plus glutamine and the OAA level was measured at 8 hpi. **(G)** VACV infection increases the ATP levels in HFFs. HFFs were infected with MOI of 2 of WT-VACV or vΔVGF (VACV with VGF gene deleted) in medium containing glucose and glutamine. The ATP levels were measured at 8 hpi by using an ATP assay kit. **(H)** TCA Cycle activity is important for VACV replication. HFFs infected with WT VACV at MOI of 2 or 0.1 in media with glucose plus glutamine in the presence or absence of 50 μM Enasidenib. VACV titers measured at 24 and 48 hpi for MOI 2 and 0.1 respectively using a plaque assay. **(I)** Enasidenib treatment has minimal effect on HFF viability. HFFs were treated with 50 μM Enasidenib in medium with glucose plus glutamine. Cell viability measured by a trypan blue assay at 48 h post treatment. Error bars represent the standard deviation of at least three biological replicates. ns, P > 0.05; *, P ≤ 0.05; **, P ≤ 0.01; ****, P ≤ 0.0001.

Next, we closely investigated the levels of the TCA cycle intermediates as it is the central hub of cellular metabolism (**Fig 2A)**, and a global metabolic reprogramming likely involved the alteration of the TCA cycle intermediates. Notably, at 8 hpi, most of the TCA cycle intermediates are significantly higher in VACV-infected cells than in mock-infected cells, in both glucose plus asparagine and glucose only conditions (the succinate levels were similar in mock- and VACV-infected cells) (**Fig 2B**). At 16 hpi, although we still observed the general trend that the TCA intermediate levels increased in VACV-infected cells, the elevation levels decreased (**S2 Fig**), again suggesting that the elevation of the metabolites occurred at an earlier time and the metabolites were consumed at the later time of infection. Interestingly, the level of glutamate, whose biosynthesis can be fed by the TCA cycle intermediate, α-ketoglutarate [39], increased significantly in VACV infected cells in the absence of glutamine (**Fig 2C)**. Together, these results reveal the enhanced levels of the TCA cycle-related metabolites during VACV replication.

To further validate the findings of metabolic profiling, we measured the citrate level as it is the first molecule of the TCA cycle. Using a citrate assay kit, we confirmed that the citrate level significantly increased by approximately 3.3- and 3.2-fold in VACV-infected HFFs cultured in media containing either glucose only or glucose plus asparagine, respectively (**Fig 2D**). Remarkably, we also observed a similar increase of the citrate level in VACV-infected HFFs cultured in medium containing glutamine and glucose **(Fig 2E**), indicating the elevation of citrate in the presence of exogenous glutamine. The upregulation of citrate could be observed at 2 hpi (**Fig 2E**). The level of oxaloacetate (OAA), another critical metabolite of the TCA cycle and citrate metabolism (**Fig 2A**) increased by two-fold at 8 hpi (**Fig 2F**). Interestingly, in the metabolic profiling in the presence of glucose and glutamine, Fontaine et al. found a 1.49 and 1.37-fold increase of citrate levels upon VACV infection at 4 and 8 hpi, respectively, although it is not statistically significant [12]. Most of the other detected TCA cycle intermediates were also moderately (although not significantly) upregulated by up to 1.37-fold at 8 hpi [12]. Taken together, our findings corroborate that VACV infection elevates the steady-state levels of TCA cycle intermediates, which can provide metabolic foundations to modulate TCA cycle-related activities and biomolecule synthesis.

Previous work from multiple groups demonstrated that VACV promotes oxygen consumption and ATP production in different cell types [14,15,40], indicating an enhanced TCA cycle activity. We examined if VACV infection increases ATP production in HFFs and observed a significant, although not as high as in HeLa cells [15,40], increase in ATP production after VACV infection (**Fig 2G**). These findings indicate a biologically relevant activity of the elevated TCA cycle intermediate levels. To further examine if TCA cycle activity is important for VACV replication, we treated HFFs with Enasidenib (targeting the enzyme isocitrate dehydrogenase 2 of the TCA cycle). We observed a significant decrease of VACV replication (39- and 83-fold decrease at the MOI of 2 and 0.1 respectively) (**Fig 2H**) upon Enasidenib treatment at a concentration that did not alter cell viability (**Fig 2I**). These results indicate an essential role of high TCA cycle activity in VACV replication.

### VACV infection reprograms TCA cycle-related metabolism

**Fig 3:**
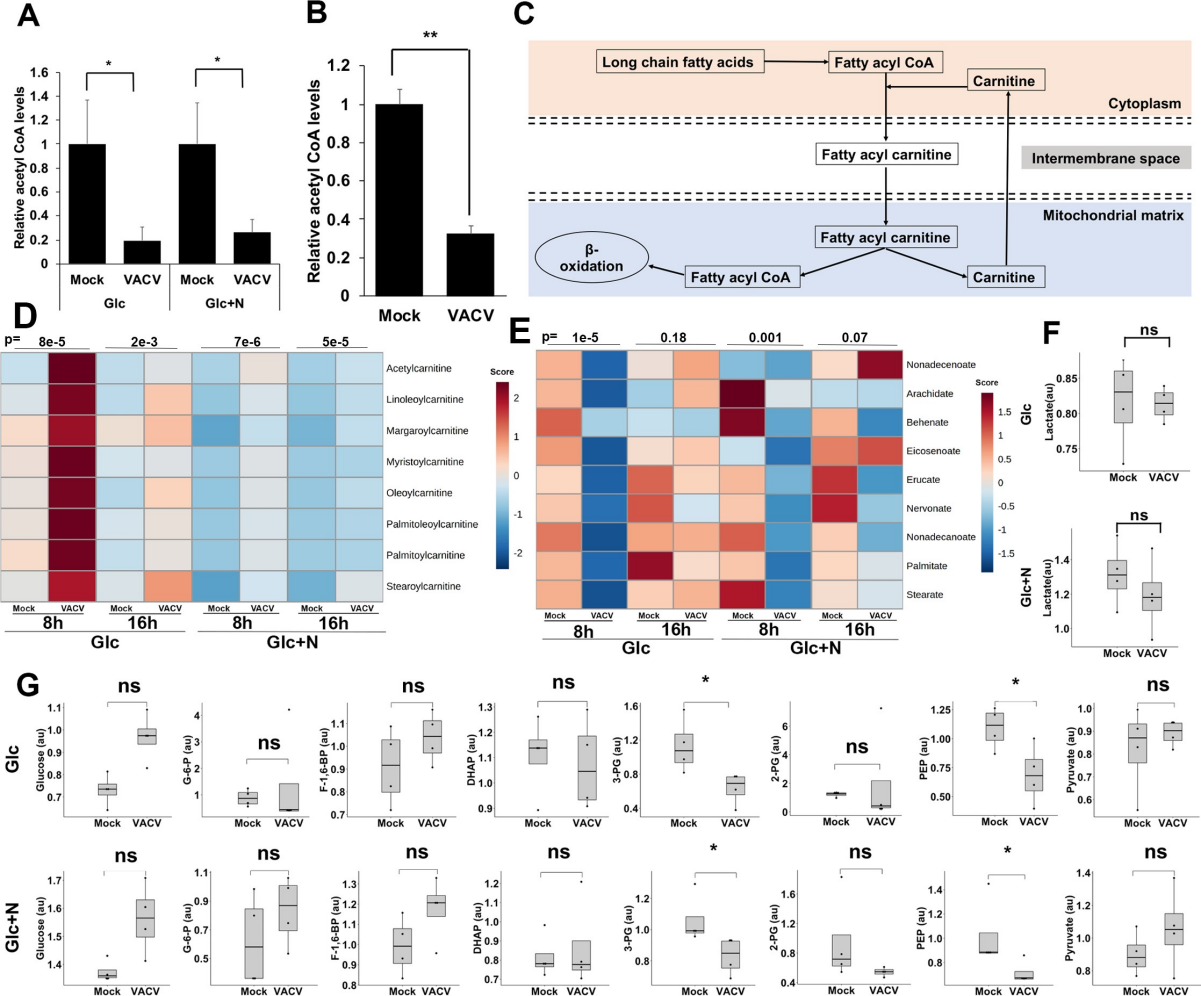
VACV infection alters the TCA cycle-related metabolism. **(A)** A decrease in Acetyl-CoA upon VACV infection in HFFs cultured in media without glutamine. The level of Acetyl-CoA at 8 hpi in the metabolic profiling of Fig 1A was shown. **(B)** VACV infection decreases the level of acetyl CoA in HFFs cultured in medium containing glutamine. HFFs infected with WT VACV at an MOI of 2 in media with glucose plus glutamine and the Acetyl-CoA level was measured at 8 hpi using an Acetyl-CoA assay kit. **(C)** A simplified overview of carnitine metabolism in β-oxidation. The long-chain fatty acids are acylated and then carnitylated by carnitine palmitoyltransferase system, which is then transported into the mitochondrial matrix for β-oxidation to fuel the TCA cycle. **(D)** VACV infection increases the levels of carnitine-conjugated fatty acids. The metabolic profiling data of fatty acyl carnitines in VACV-infected HFFs (Supplementary File S2) was uploaded to the MetaboAnalyst tool and then a hierarchically clustered heatmap was generated using Ward’s minimum variance and Euclidean distance measure. Color keys indicate the levels of different metabolites; blue: lowest, red: highest. The number on top of the plots represent the p-values comparing the average levels of indicated metabolites levels in mock- and VACV-infected HFFs **(E)** The levels of long-chain fatty acids are reduced in VACV-infected HFFs. The metabolic profiling data of long-chain fatty acids in VACV-infected HFFs (Supplementary File S2) was processed as in Fig 3D. **(F)** VACV infection does not affect the level of lactate. The level of lactate in HFFs infected with MOI-3 of WT-VACV in media with glucose (Glc) or glucose plus asparagine (Glc+N) at 8 hpi was determined by global metabolic profiling in Fig 1A. **(G)** The glycolysis intermediates are either unaffected or reduced by VACV infection. The levels of glycolysis intermediates in HFFs infected with MOI-3 of WT-VACV in medium with glucose (Glc) or glucose plus asparagine (Glc+N) at 8 hpi as determined by global metabolic profiling in Fig 1A. Error bars represent the standard deviation of at least three biological replicates. ns, P > 0.05; *, P ≤ 0.05; **, P ≤ 0.01; ***, P ≤ 0.001; ****, P ≤ 0.0001.

Acetyl-CoA is an indispensable player in citrate biosynthesis and breakdown (**Fig 2A**). At 8 hpi we observed a significant 81% and 74% decrease of the Acetyl-CoA levels upon VACV infection through metabolic profiling in medium with glucose or glucose plus asparagine, respectively (**Fig 3A**). The level of Acetyl-CoA was still significantly reduced at 16 hpi (**S3 Fig**). Notably, there was a similar significant reduction in the Acetyl-CoA level in culture medium containing glutamine (**Fig 3B**). In the absence of glutamine, glucose and fatty acids are two other major carbon sources of the TCA cycle (**Fig 2A**) [41]. These findings suggest that Acetyl-CoA is heavily consumed, or its synthesis is suppressed during VACV infection. Although the lipid species are both up and down-regulated (**S1A and S1B Fig**), the fatty acyl-carnitines, which are used up in β-oxidation to be converted to acetyl-CoA after being transported to the mitochondria [42], significantly increased in the metabolic profiling **(Fig 3C and 3D).** Interestingly, those non-carnitine-conjugated long-chain fatty acid levels decreased significantly at 8 hpi (**Fig 3E**). Although not statistically significant, the metabolic profiling in the presence of glutamine by Fontaine et al. showed a moderate increase in all the detected carnitine-conjugated fatty acids at 8 hpi [12]. These results suggest the metabolism of fatty acids is significantly reprogramed towards an enhanced levels of carnitine conjugation during VACV infection. Analysis of the glycolysis products by metabolic profiling in glutamine-depletion conditions indicated that several essential glycolysis products decreased in VACV-infected HFFs (**Figs 3G and S4**). The levels of lactate were similar in both growth conditions upon VACV infection (**Figs 3F and S5**), suggesting that VACV infection did not utilize glucose to undergo anaerobic respiration. In the presence of glutamine, the levels of most of the glycolysis intermediates were not significantly altered in the metabolic profiling of VACV-infected HFFs carried out by Fontaine et al [12]. Because VACV infection did not increase the level of glucose, the lowered glycolysis could suggest two possibilities. First, glycolysis products were heavily consumed to feed the TCA cycle in VACV-infected cells under glutamine-depletion conditions. Second, glycolysis was down-regulated during VACV infection, which would suggest a more important role of fatty acids to feed the TCA cycle. Overall, these results reveal a systematic reprogramming of TCA cycle-related metabolism during VACV infection.

### Inhibition of glycolysis or fatty acid β-oxidation abolishes citrate level increase during VACV infection

**Fig 4:**
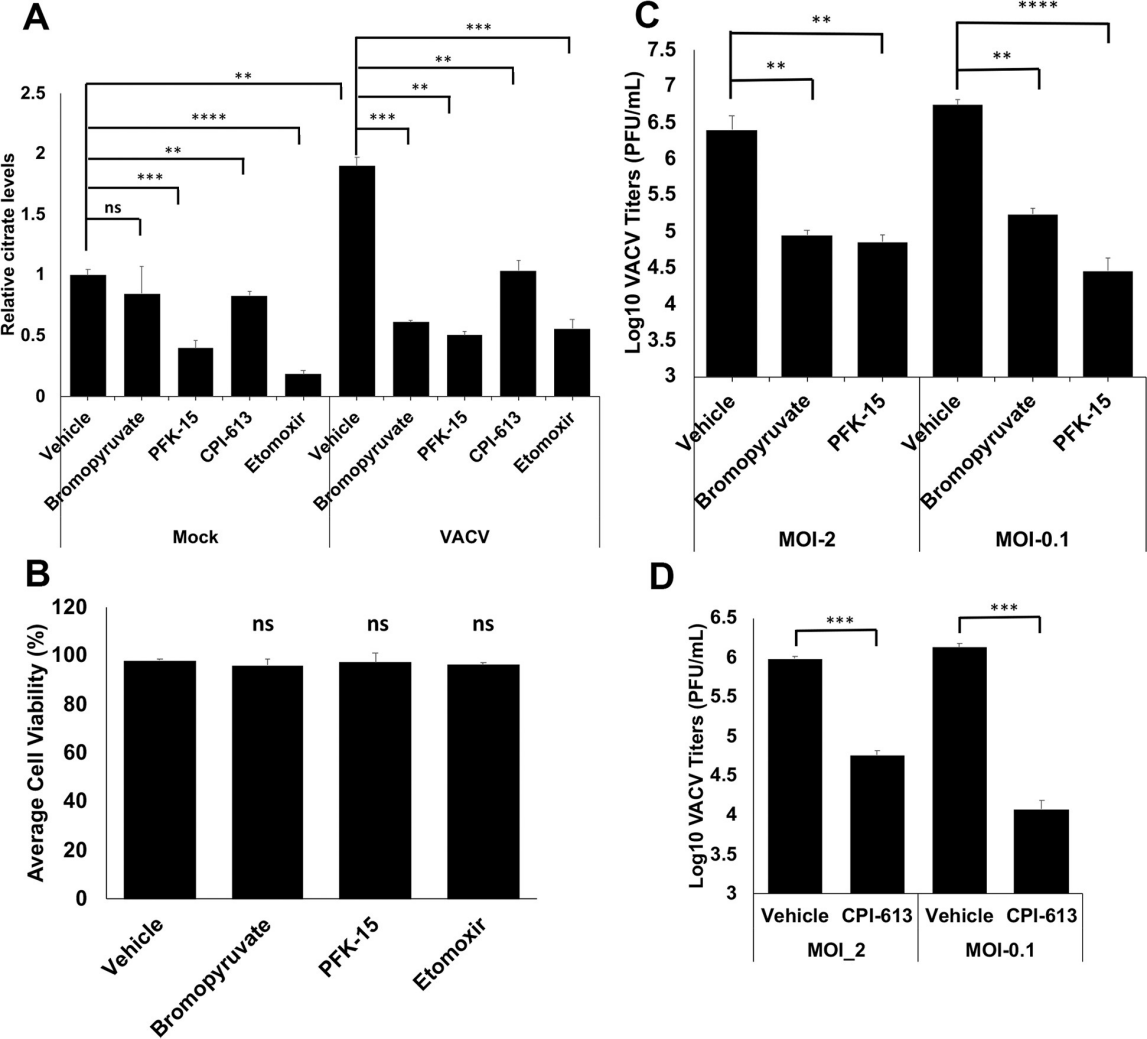
Both Glycolysis and β-oxidation contribute towards the citrate level enhancement during VACV infection. **(A)** Inhibition of glycolysis and fatty acid oxidation reduces the increase of citrate levels during VACV infection. HFFs were mock-infected or infected with WT-VACV at an MOI of 5 in medium with glucose plus glutamine in the presence or absence of 50 μΜ bromopyruvate, 50 μM PFK-15, 100 μΜ of CPI-613, and 50 μΜ etomoxir. Citrate levels measured at 4 hpi using a citrate assay kit. **(B)** HFFs treated with indicated chemicals at a concentration as listed in Fig 4A in medium with glucose plus glutamine. Cell viability measured by a trypan blue assay at 48 h post treatment. **(C)** Glycolysis inhibition suppresses VACV replication. HFFs infected with WT VACV at an MOI of 2 (for 24 h) or MOI of 0.1 (for 48 h) in medium with glucose plus glutamine with or without 50 μΜ bromopyruvate, 50 μM PFK-15. Virus titers measured by a plaque assay. **(D)** Inhibition of pyruvate dehydrogenase and α-ketoglutarate dehydrogenase reduces VACV titers. HFFs infected with WT VACV at an MOI of 2 (for 24 h) or MOI of 0.1 (for 48 h) in medium with glucose plus glutamine in the presence or absence of 100 μΜ CPI-613. Virus titers were measured by a plaque assay. Error bars represent the standard deviation of at least three biological replicates. ns, P > 0.05; **, P ≤ 0.01; ***, P ≤ 0.001; ****, P ≤ 0.0001.

Our metabolic profiling data could not answer the question if glycolysis or β-oxidation individually contributes to the increase of citrate levels during VACV infection. We used several inhibitors targeting glycolysis and β-oxidation to assess their effects on citrate levels during VACV infection. As can be seen in **Fig 4A**, bromopyruvate (targeting the first enzyme, hexokinase, of glycolysis [43]), PFK15 (targeting the rate-limiting enzyme, 6-phosphofructo-2-kinase, of glycolysis [44]), CPI-613 (targeting pyruvate dehydrogenase and α-ketoglutarate dehydrogenase [45]), and etomoxir (targeting carnitine palmitoyltransferase-1 of β-oxidation [46]) all decreased the citrate levels in VACV-infected HFFs, at the concentrations that did not affect HFF viability in the absence of infection (**Fig 4B**). In uninfected cells, PFK-15 and etomoxir, but not bromopyruvate and CPI-613, also decreased the citrate levels (**Fig 4A**). It has been reported that etomoxir treatment significantly suppresses VACV replication [14]. Here we observed significant reduction of VACV replication by bromopyruvate, CPI-613 and PFK-15 treatment, respectively (**Fig 4C and 4D**). These findings indicate that both glycolysis and β-oxidation contribute significantly to the increased citrate levels during VACV infection.

### VGF gene deletion abolishes the elevation of citrate level during VACV infection

**Fig 5:**
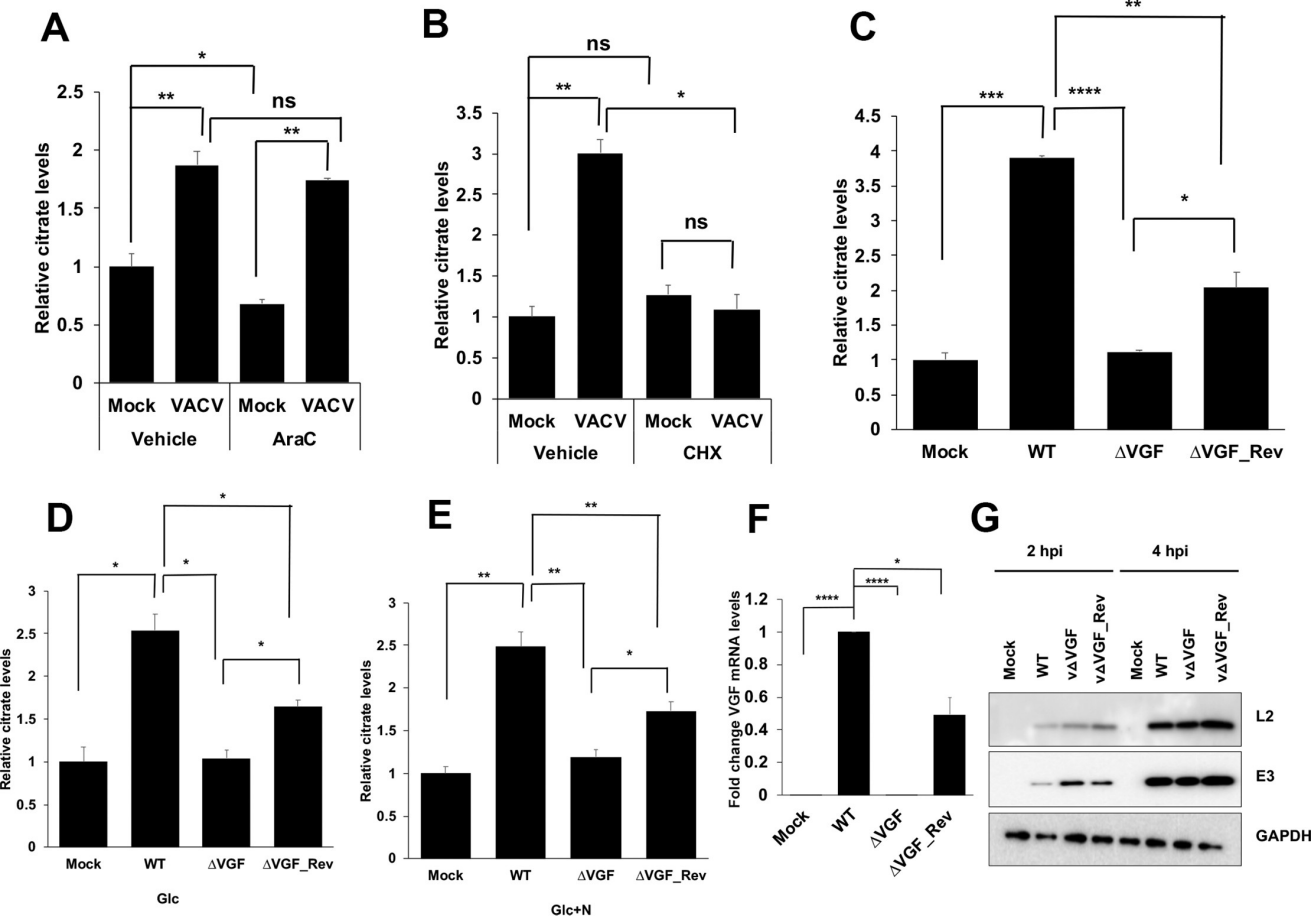
VACV growth factor (VGF) deletion abolishes the elevation of citrate level during viral infection. **(A)** Inhibition of DNA synthesis does not inhibit the increased citrate level upon VACV infection. HFFs were infected with VACV at an MOI of 5 in medium with glucose plus glutamine in the presence or absence of 40 μg/mL AraC. Citrate level was measured at 8 hpi. **(B)** Inhibition of protein synthesis reduces citrate level in VACV-infected HFFs. HFFs were infected with VACV at an MOI of 5 in medium with glucose plus glutamine in the presence or absence of 100 μg/mL Cycloheximide. Citrate level was measured at 2 hpi. **(C-E)** VGF is required for the elevation of citrate level during VACV infection. **(C)** HFFs were infected with either WT-VACV or vΔVGF or a VGF revertant vΔVGF_Rev at an MOI of 5 in medium with glucose plus glutamine. Citrate level was measured at 4 hpi. **(D)** HFFs were infected with indicated viruses at an MOI of 5 in medium with glucose only (Glc). Citrate level was measured at 4 hpi. **(E)** HFFs were infected with indicated viruses at an MOI of 5 in with glucose + asparagine (Glc+N), and citrate level was measured at 4 hpi. **(F)** VGF mRNA expression in WT-VACV, vΔVGF, and vΔVGF_Rev. RNA was extracted from HFFs infected with indicated viruses at an MOI of 5 for 1 h in medium with glucose plus glutamine, and reverse transcription-quantitative PCR (qRT-PCR) analysis was performed. **(G)** VGF deletion does not affect the levels of other VACV early proteins. HFFs infected with indicated viruses at an MOI of 5. Western blotting analysis was performed at indicated time post infection to measure the levels of VACV E3 and L2 proteins. Error bars represent the standard deviation of at least three biological replicates. ns, P > 0.05; *, P ≤ 0.05; **, P ≤ 0.01; ***, P ≤ 0.001; ****, P ≤ 0.0001.

Our previous study indicated that VACV replication was suppressed at a late replication stage in medium containing glucose only, without glutamine/asparagine [38]. The upregulation of citrate and other metabolites in HFFs cultured in glucose only medium suggests an early event of VACV replication is responsible. We further tested if viral DNA replication inhibition affected the upregulation of citrate level in VACV-infected cells using AraC, a well-established inhibitor of DNA replication but not viral early protein synthesis [47]. AraC treatment did not inhibit the increase in citrate level upon VACV infection (**Fig 5A**), indicating an event prior to viral DNA replication could stimulate the citrate level. However, treatment with cycloheximide (CHX), a well-known inhibitor of mRNA translation [48], abolished the citrate level’s increase upon VACV infection. In contrast, cycloheximide treatment did not affect the citrate level in uninfected HFFs (**Fig 5B**). These results suggest that early VACV protein expression is required to enhance the citrate level upon VACV infection.

We tested several viral early genes and found that VGF is needed for VACV to increase the citrate level. We generated a recombinant VACV with both copies of the VGF gene deleted (vΔVGF). We also made a VGF revertant VACV (vΔVGF_Rev), with one copy of the VGF gene under its natural promoter inserted at a different locus of the original VGF gene in the central region of the viral genome. VGF was known to be critical for VACV replication and virulence in infected mice [34,35]. While the role of VGF in cultured cells was less prominent, we observed a significant 4.2-fold yield reduction of vΔVGF in HFFs (**S6A Fig**), similar to what had been observed in BSC40 cells [32]. While VACV infection of HFFs could not form clear and measurable plaques, we observed significantly smaller plaques of vΔVGF in BS-C-1 cells (**S6B Fig**), similar to what had been observed previously in BSC40 cells [32]. Remarkably, while WT-VACV infection resulted in a significant increase in citrate level, the deletion of VGF rendered VACV unable to enhance the level of citrate upon infection, regardless of the culture medium contents (**Fig 5C–5E**). Interestingly, vΔVGF_Rev partially recused the citrate level enhancement, consistent with that the VGF mRNA level in the vΔVGF_Rev was approximately 50% of that in WT-VACV infected cells (**Fig 5F**). Moreover, VACV early gene expression was not affected by the deletion of VGF, evidenced by similar levels of two viral early proteins, E3 and L2, in WT, vΔVGF, and vΔVGF_Rev infected HFFs (**Fig 5G**), further corroborating that the reduced level of citrate in vΔVGF-infected cells was due to a lack of VGF expression. Interestingly, we also observed a reduction of ATP level in vΔVGF-infected than in WT VACV-infected HFFs (**Fig 2G**). Overall, our results demonstrate VACV elevation of the citrate level depends on VGF expression.

To investigate if VGF is sufficient for the increased level of citrate, we used a synthetic peptide of processed VGF to treat HFFs. However, we could not observe a rescue of citrate level (**S7 Fig**). The finding is not conclusive as it is not clear the failure to elevate the citrate level by this peptide was due to VGF alone is not sufficient or the synthetic peptide is not fully and biologically active. Further studies using different approaches are needed.

### EGFR, MAPK, and STAT3 signaling pathways are needed for citrate level increase in VACV-infected cells

**Fig 6:**
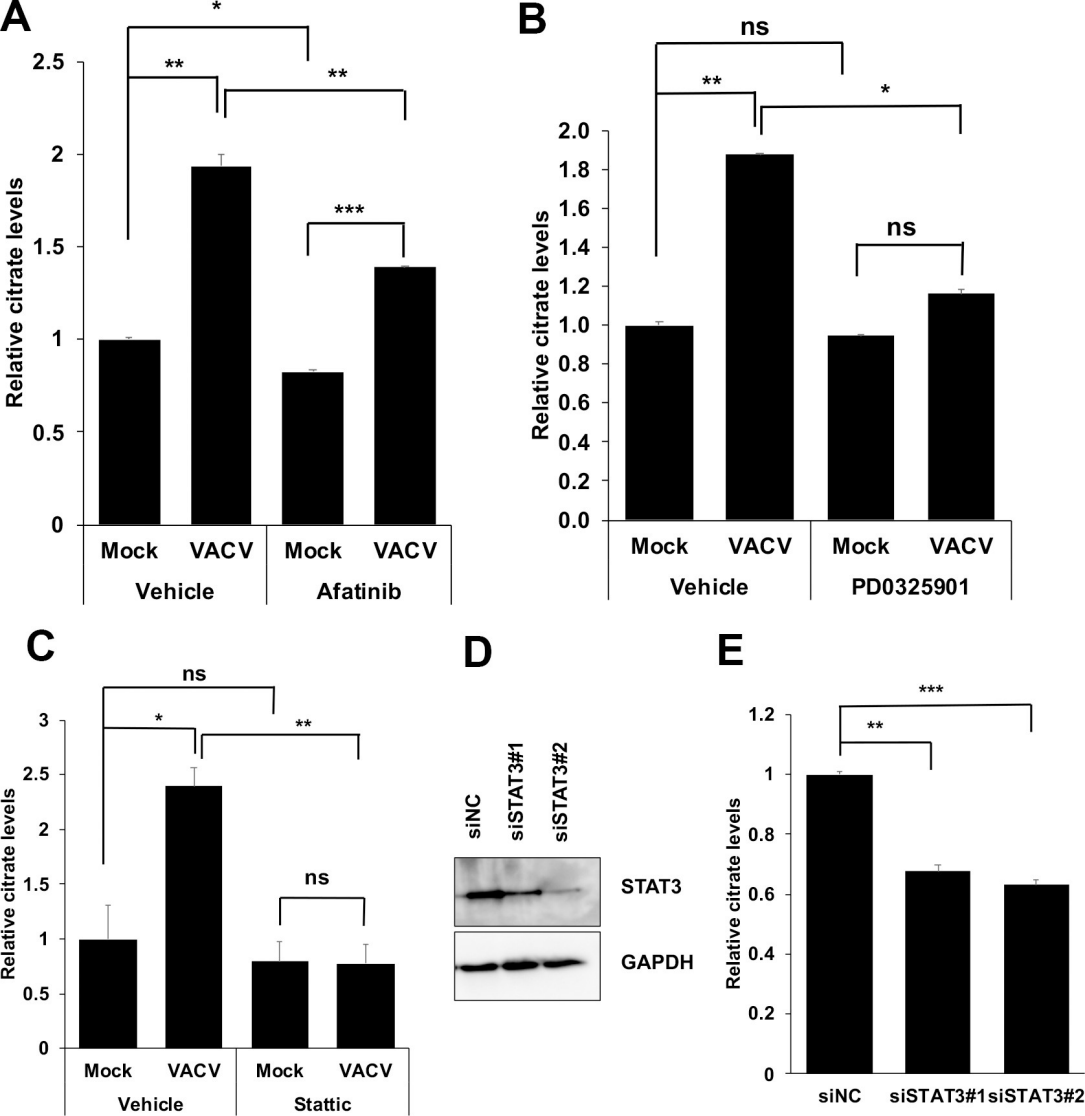
Inhibition of the STAT3 pathway and its upstream signaling decreases citrate levels during VACV infection. **(A)** Inhibition of the EGFR pathway decreases the citrate level in VACV-infected HFFs. HFFs were infected with WT VACV at an MOI of 5 in the presence or absence of 3 μM afatinib. The citrate level was measured at 4 hpi. **(B)** Inhibition of the MAPK pathway decreases the citrate level during VACV infection. HFFs were infected with WT VACV at an MOI of 5 in the presence or absence of 20 μM PD0325901. The citrate level was measured at 2 hpi. **(C)** Inhibition of the STAT3 pathway decreases the citrate level in VACV-infected cells. HFFs were infected with VACV at an MOI of 5 in the presence or absence of 3 μM stattic. The citrate level was measured at 4 hpi. **(F)** siRNA-mediated knockdown of STAT3. HFFs were transfected with a negative control siRNA or two specific siRNA targeting STAT3 for 48 h. Western blotting analysis was performed to measure the level of STAT3. **(G)** siRNA-mediated knockdown of STAT3 decreases citrate level during VACV infection. HFFs were transfected with indicated siRNAs for 48 h and then infected with an MOI of 5 of VACV for 4 h, and the citrate level was measured. All the infections were performed in media with glucose plus glutamine. Error bars represent the standard deviation of at least three biological replicates. ns, P > 0.05; *, P ≤ 0.05; **, P ≤ 0.01; ***, P ≤ 0.001.

VGF is homologous to cellular EGF that activates the EGFR and MAPK pathways [32,49]. We hypothesized that VGF-mediated cell signaling is required for the increasing citrate level upon VACV infection. We first tested the effect of afatinib, an irreversible inhibitor of the EGFR pathway on citrate metabolism [50]. We found that VACV infection resulted in an increase in citrate levels, while EGFR inhibition with afatinib at a concentration that did not affect cell viability reduced the increase in the citrate level upon VACV infection **(Figs 6A and S8A).** Although it also decreased the citrate level in uninfected controls, the reduction was only about 18%. Afatinib treatment significantly reduced VACV titer by 43-fold at the same concentration (**S8B Fig**), agreeing with a previous study on the effect of EGFR inhibitors on VACV replication [51]. We then tested the effect of inhibiting the MAPK pathway on citrate level using PD0325901, a selective inhibitor of MAPK/ERK pathway [52]. While VACV infection resulted in an increase in citrate level in vehicle-treated cells, PD0325901 treatment significantly reduced the citrate level in VACV infected cells to the level comparable to uninfected cells (**Fig 6B**). Furthermore, MAPK pathway inhibition resulted in a 67-fold reduction of VACV titer (**S9A Fig**), at a concentration that did not affect the viability of HFFs (**S9B Fig**), consistent with an earlier study [49]. It is worth noting that both EGFR and MAPK pathways are activated by VGF during VACV infection [32,49,53]. Therefore, our results indicate that the EGFR and MAPK signaling pathways are required for the upregulation of the citrate level during VACV infection.

One downstream signaling molecules of the EGFR-MAPK axis is the STAT3, as EGFR induced MAPK pathway is a major upstream activator of non-canonical STAT3 phosphorylation at serine 727 (S727) [54,55]. Notably, stattic, an inhibitor of STAT3 activation [56], significantly reduced the increase in citrate level in VACV-infected cells but not in uninfected cells (**Fig 6C**). Chemical inhibition of the STAT3 pathway by stattic resulted in a 177-fold reduction in VACV titers in HFFs (**S10A Fig**), consistent with our results in other cell types and an unbiased screening of compounds of VACV inhibitors [57]. Stattic treatment did not affect HFF viability at the same concentration (**S10B Fig**), suggesting that STAT3 signaling is also required for VACV-induced citrate level increase. Further supporting the critical role of STAT3 signaling in citrate level upregulation during VACV infection, specific siRNA treatment significantly decreased the citrate level during VACV infection (**Fig 6F and 6G**) without affecting the HFF viability (**S10C Fig**).

### VACV infection stimulates non-canonical STAT3 activation in a VGF-dependent manner

**Fig 7:**
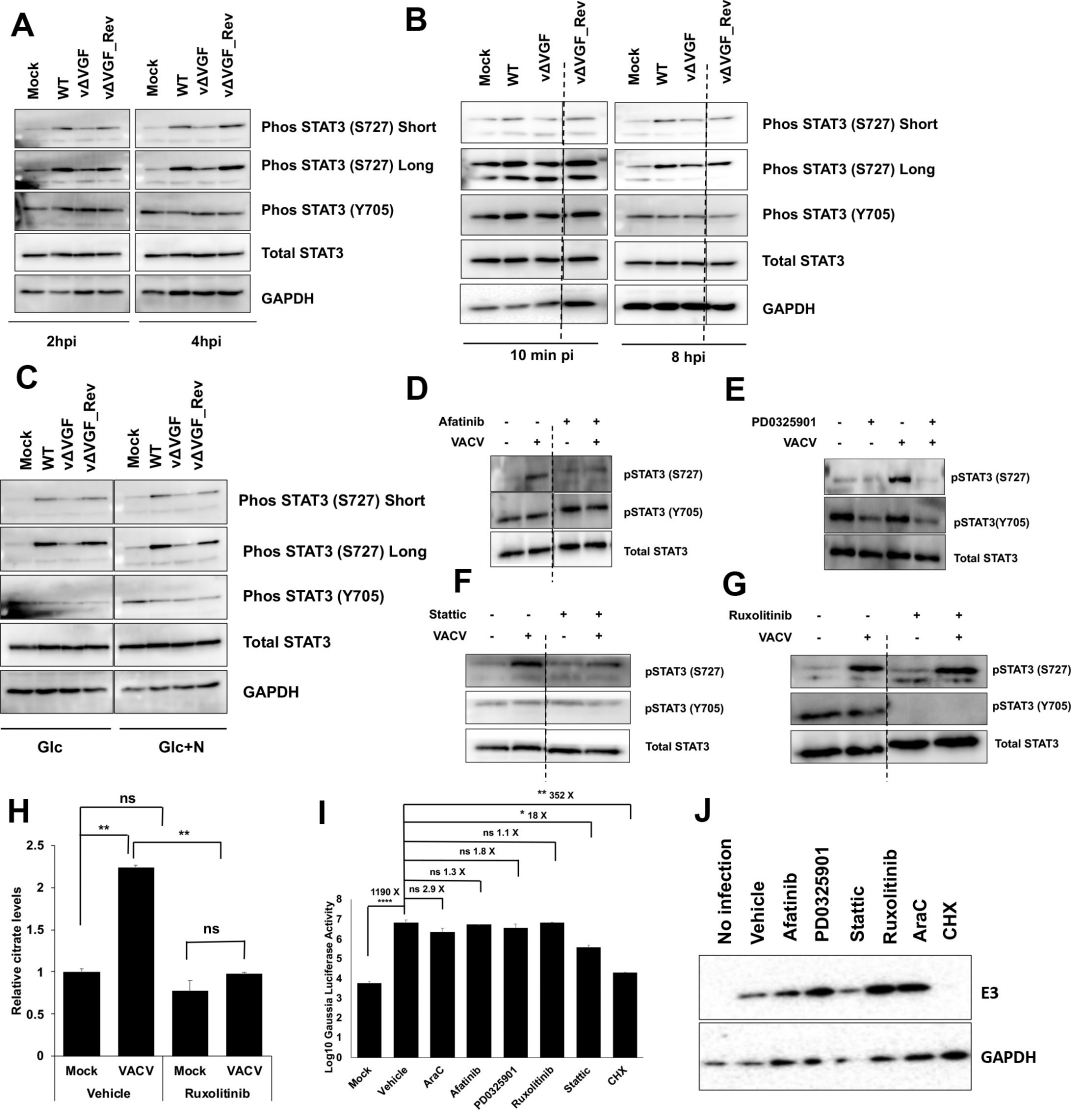
VACV infection induces non-canonical STAT3 phosphorylation at S727 in a VGF-dependent manner. **(A)** VACV VGF is indispensable to activate STAT3 (S727) phosphorylation. HFFs infected with indicated viruses at an MOI of 5 for the indicated time. Western blotting analysis was performed to measure the levels of various forms of STAT3. (**B)** Upregulation of STAT3 S727 phosphorylation starts early during VACV infection. HFFs infected with indicated viruses at an MOI of 5. The samples were collected at 10 min post-infection and 8 hpi, respectively, followed by Western blotting analysis. **(C)** VACV activates STAT3 (S727) phosphorylation in the absence of glutamine in the medium. HFFs were infected with indicated viruses at an MOI of 5 in medium with glucose only (Glc) or with glucose+asparagine (Glc+N). Protein levels were detected by performing a Western blotting analysis at 4 hpi. **(D)** Inhibition of the EGFR pathway decreases STAT3 S727 phosphorylation in VACV infected cells. HFFs were infected with MOI of 5 of WT-VACV with or without 3 μM afatinib treatment. Western blotting analysis was performed at 4 hpi to test the levels of different forms of STAT3 protein. **(E)** Inhibition of the MAPK pathway decreases both Y705 and S727 phosphorylation. HFFs were infected with MOI of 5 of VACV in medium with or without 20 μM PD0325901 treatment. Western blotting analysis was performed at 2 hpi to detect the levels of different forms of STAT3 protein. **(F)** Stattic treatment inhibits S727 phosphorylation. HFFs were infected with MOI of 5 of WT VACV with or without 3 μM stattic. At 4 hpi, Western blotting analysis was performed to detect the levels of different forms of STAT3 protein. **(G)** STAT3 S727 phosphorylation is independent of the JAK1/2 pathway. HFFs were infected with an MOI of 5 of VACV in medium with or without 5 μM ruxolitinib treatment. Western blotting analysis was performed at 4 hpi to measure different protein levels. **(H)** Ruxolitinib treatment decreases the induction of citrate level upon VACV infection. HFFs were infected with WT VACV at an MOI of 5 in the presence or absence of ruxolitinib treatment. The citrate level was measured at 4 hpi. **(I)** Effects of inhibition of STAT3 and its upstream signaling pathways on VACV early protein expression. HFFs infected with WT VACV at an MOI of 2 in the presence or absence of 3 μM afatinib, 20 μM PD0325901, 3 μM stattic, 5 μM ruxolitinib, 40 μg/mL AraC, or 100 μg/mL cycloheximide. The levels of VACV E3 protein was measured at 2 hpi by a Western blotting analysis. **(J)** Effects of inhibition of STAT3 and its upstream signaling pathways on VACV early protein levels. HFFs infected at an MOI of 2 with a recombinant VACV expressing *Gaussia* luciferase under virus early VGF promoter in the presence or absence of 3 μM afatinib, 20 μM PD0325901, 3 μM stattic, 5 μM ruxolitinib, 40 μg/mL AraC, or 100 μg/mL cycloheximide. Early gene expression was measured by a *Gaussia* luciferase activity assay kit at 2 hpi. All experiments were performed in media with glucose plus glutamine unless otherwise stated. Error bars represent the standard deviation of at least three biological replicates. ns, P > 0.05; *, P ≤ 0.05; **, P ≤ 0.01; ****, P ≤ 0.0001. The blots were from different lanes on the same gel and the dashed lines indicate that some non-relevant lanes were removed.

STAT3 can be phosphorylated at tyrosine 705 position (Y705) (induced mainly by JAK1/2 pathway) and at serine 727 (S727) (induced mainly by MAPK pathway); known as the canonical and non-canonical phosphorylation, respectively [55,58]. We analyzed STAT3 phosphorylation in HFFs infected with WT or vΔVGF or vΔVGF_Rev VACV at 2 and 4 hpi, using medium containing glucose and glutamine. VACV infection selectively upregulated the non-canonical STAT3 phosphorylation at the S727 (**Fig 7A**). Notably, the deletion of VGF abolished STAT3 S727 phosphorylation, which could be rescued by the VGF revertant mutant (**Fig 7A**). In contrast, the canonical Y705 phosphorylation of STAT3 did not increase upon VACV infection (**Fig 7A**). The VGF dependent upregulation of the non-canonical STAT3 pathway was seen as early as 10-minute post-infection and could still be observed at 8 hpi (**Fig 7B**). The early stimulation of STAT3 S727 phosphorylation is consistent with the fact that VGF is an early gene and it starts to be expressed immediately after VACV enters the cells [4]. Similar results were found when using medium containing no glutamine (**Fig 7C**), indicating the VGF dependent phosphorylation of STAT3 at S727 can be achieved in a glutamine-independent manner.

Next, we determined if the EGFR and MAPK signaling is needed for STAT3 phosphorylation at S727 during VACV infection. Afatinib treatment noticeably decreased the S727 phosphorylation in VACV infected cells. However, it did not affect Y705 phosphorylation (**Fig 7D**), indicating a pivotal role of the EGFR pathway in non-canonical STAT3 activation during VACV infection. MAPK inhibitor, PD0325901, inhibited S727, and Y705 STAT3 phosphorylation, the former was more evident in VACV-infected cells (**Fig 7E**). The results suggest that the PD0325901 also inhibited STAT3 Y705 phosphorylation, likely via signaling crosstalk. There is no S727 specific STAT3 inhibitor available. The STAT3 inhibitor, stattic, partially inhibited the VACV infection-mediated increase of S727 phosphorylation, but with no noticeable effect on Y705 phosphorylation (**Fig 7F**). The latter was not changed much upon VACV infection. These results demonstrate the requirements of VGF, EGFR, and MAPK in non-canonical activation of STAT3 at S727. Together with the results in **Fig 6**, the results also indicate the indispensable roles of VGF, EGFR, MAPK, and STAT3 in citrate level elevation during VACV infection. While the non-canonical STAT3 signaling is activated to elevate the citrate level, the canonical pathway is not stimulated by VACV infection.

We examined if the JAK-STAT3 axis that phosphorylates Y705 is required for citrate induction during VACV infection, although it is not further activated by VACV infection. Ruxolitinib, an inhibitor of JAK1/2 that is the primary upstream activator of STAT3 Y705 phosphorylation [59], did not affect S727 phosphorylation but inhibited the Y705 phosphorylation in both uninfected and VACV infected cells (**Fig 7G**). Ruxolitinib deceased citrate level in the uninfected cells by 23% while it significantly reduced the induction by 55% in VACV infection (**Fig 7H**) without affecting HFF viability (**S11 Fig**). This result suggests that the canonical STAT3 activity is also required for VACV elevation of the citrate level. Furthermore, viral early proteins were still expressed with the treatments with EGFR, MAPK, STAT3, or JAK1/2 inhibitors. EGFR, MAPK, and JAK1/2 inhibitors had little effects on viral early protein levels, evidenced by the expression of a viral early protein E3 and a reporter VACV with *Gaussia* luciferase expression under the control of the VACV early VGF gene promoter [38] (**Fig 7I and 7J**). While the STAT3 inhibitor (stattic) treatment decreased VACV early protein levels, considerable amounts of viral early proteins were still expressed (**Fig 7I and 7J**). The result suggests that stattic also suppresses VACV replication at or prior to viral gene expression steps.

## Discussion

**Fig 8:**
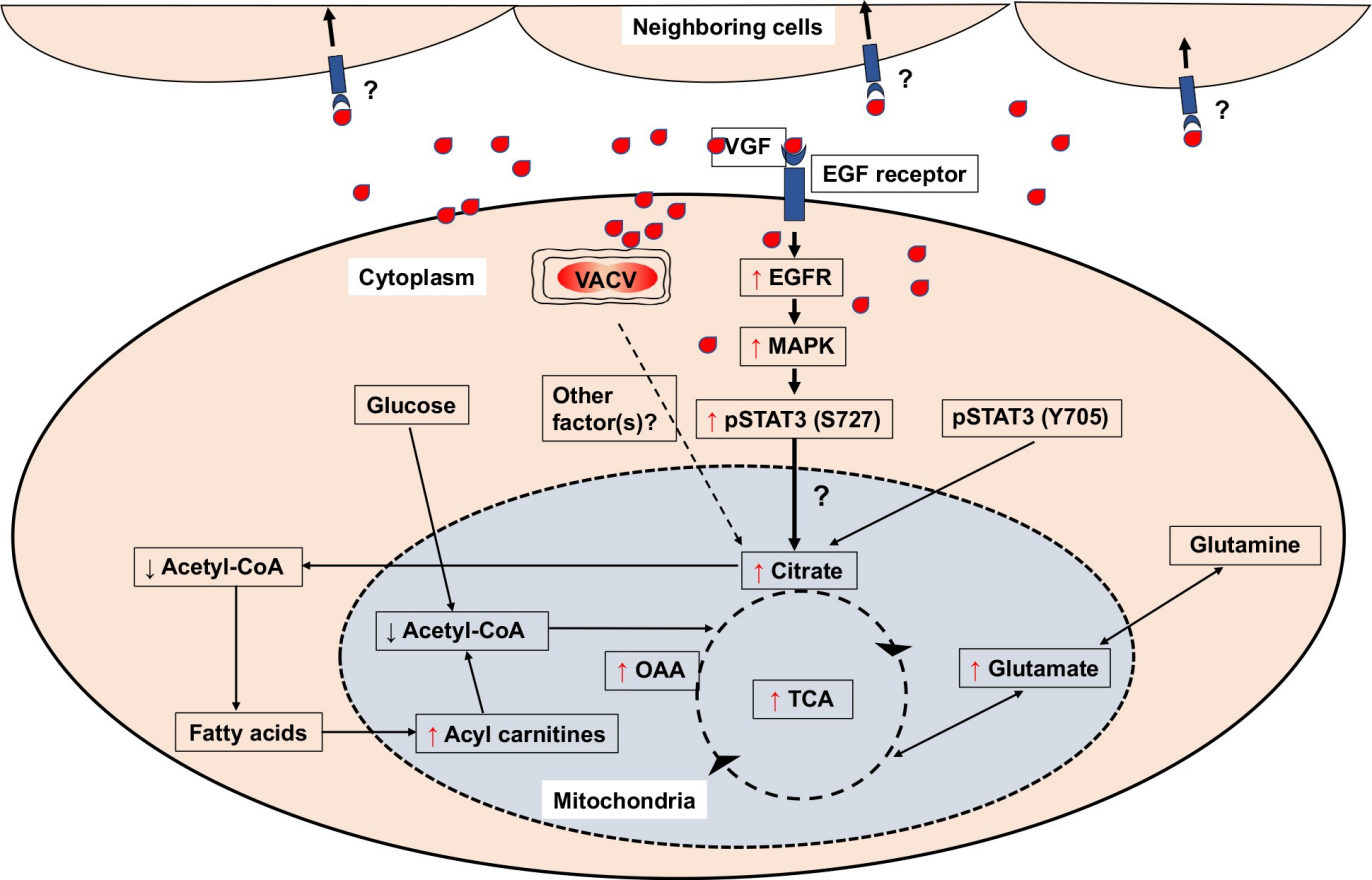
Proposed model by which VACV infection promotes the TCA cycle. VACV infection enhances the levels of TCA cycle intermediates and related products. Upon VACV infection, the levels of Acetyl-CoA decrease, while the levels of fatty acyl carnitines (key metabolites for β-oxidation of fatty acids) increase. The increase in the level of citrate can be attributed to the VACV VGF mediated upregulation of non-canonical STAT3 phosphorylation at S727 via EGFR and MAPK pathways. Although not upregulated by VACV, the Y705 phosphorylation of STAT3 is also important for enhancing citrate level. It is unclear if additional viral factors are also required to elevate the TCA cycle and if VGF alone can exert the function in uninfected cells. Red upward arrows indicate increase and black downward arrows indicate decrease of indicated intermediates.

In this study, we discovered a novel VACV metabolic reprogramming strategy that elevates the intermediates of the TCA cycle, the cellular metabolic hub. We determined the viral factor and cellular signaling pathways driving this metabolic alteration for an elevated citrate level, the first molecule of the TCA cycle. The findings lead to a model by which VACV elevates the TCA cycle intermediate levels (**Fig 8**): VACV produces VGF at an early time of infection. The VGF then stimulates the EGFR/MAPK/non-canonical STAT3 signaling axis in the infected and perhaps also in the uninfected neighboring cells to reprogram the TCA cycle and its related cellular metabolism. While the canonical STAT3 signaling is not stimulated by VACV infection, its basal activity is still required. At this point, we cannot conclude if VGF alone is sufficient to exert this effect as our data using a synthetic VGF peptide failed to elevate the citrate level in the absence of VACV infection (**S7 Fig**). Further investigations using a system more closely mimicking the natural route of VGF expression and processing in the absence of VACV infection is needed to answer this question. Moreover, the mechanistic details of the TCA cycle reprogramming and the broad impacts of the elevated TCA cycle intermediate levels are yet to be fully determined.

The steady-state levels of metabolites in cells are a net outcome of dynamic metabolism, including uptake from and secretion to extracellular space, synthesis and consumption. While our data in this study and studies from multiple groups suggest that VACV infection promotes the TCA cycle and related metabolism including oxygen consumption, ATP production, glutaminolysis [13–15], our results that VACV infection elevates the levels of citrate and other TCA cycle intermediates, and alters other related metabolites, does not give a quantitative and definitive answer on how the synthesis or consumption contributes to the final outcomes.

We have previously shown that VACV replication is fully rescued in medium containing glucose and asparagine in glutamine-depleted conditions [38,60]. This culture medium provides a unique VACV infection system to study VACV-induced manipulation of cellular metabolism in the absence of the complications caused by VACV’s upregulation of glutaminolysis [12,13]. Glucose, glutamine, and fatty acids are the three major carbon sources to feed the TCA cycle. while it was known that VACV stimulates glutaminolysis, our results using chemical inhibitors indicate that both glycolysis and β-oxidation of fatty acids are needed to increase the TCA cycle intermediates (**Fig 4**). Because of the upregulation of the carnitine-conjugated lipids, VACV may promote β-oxidation of fatty acids to elevate the TCA cycle. The metabolic profiling data support a possibility that fatty acyl-carnitines, which enter mitochondria to feed the TCA cycle, are selectively upregulated in the absence of glutamine. More mechanistic and comprehensive investigations of various branches of glycolysis and fatty acid metabolism are needed, including the modulation of the activities of key enzymes involved and the metabolic flux of the carbon by VACV infection.

The TCA cycle is at the heart of major cellular pathways for carbohydrate, lipid, and amino acid metabolism. TCA cycle intermediates and other metabolic products are the sources for the production of cellular energy and many biosynthetic precursors. The TCA cycle is also named the citric acid cycle due to its first molecule, citrate. Citrate is essential not only to drive the TCA cycle forward in the mitochondria but is also transported to the cytosol to be used for fatty acid biosynthesis [16]. Our finding that VACV infection elevates the citrate level and many other TCA cycle intermediates, but simultaneously decreases the Acetyl-CoA level, suggests that the virus has evolved to reprogram the hub of cellular metabolism to create a favorable environment for its replication. Our results can explain the chemical foundations for the oxidative phosphorylation pathway (OXPHOS) upregulation by VACV infection. The OXPHOS is the major source of cellular energy in ATP [61]. During VACV infection, there is an increase in the oxygen consumption rate (OCR), an indicator of energy metabolism [14], as well as ATP production [15,40]. Increased citrate and TCA cycle intermediates upon VACV infection also provide the substrates to upregulate the biosynthesis of other biomolecules, evidenced by the metabolic profiling indicating higher glutamate production, and carnitylated lipids during VACV infection in the absence of glutamine. Greseth et al. demonstrated that VACV replication requires *de novo* fatty acids biosynthesis [14], which requires citrate as a source of precursor.

VGF induces cell proliferative responses [29,31]. The identification of VGF as the required VACV protein to stimulate the citrate level provides the metabolic foundation of VGF’s functions in many aspects of VACV infection. Although it does not affect VACV replication in some proliferating cells, the deletion of VGF from VACV reduces VACV replication in resting cells and proliferating HFFs (**S6 Fig**). Because the metabolic level is higher in proliferating cells than in resting cells [62], the different replication phenotypes are at least partially due to different metabolic statuses in these cells. As most of the cells are in resting state in an animal, the VGF’s function to stimulate the TCA cycle intermediates could also explain the reduced replication and virulence in mice [34,35]. Since cell mobility consumes energy [63], it explains that VGF is crucial for facilitating cell motility for virus spread [32]. In addition to enhancing the motility, the secreted VGF induces EGFR in a paracrine fashion [64], which may instruct the neighboring uninfected cells to be metabolically prepared for infection.

STAT3 is a transcription factor activated by growth factors, oncogenes, and cytokines that leads to cell proliferation, migration, and differentiation, etc. [65]. While the canonical pathway of STAT3 activation with Y705 phosphorylation has been well-understood to stimulate gene transcription in cell proliferation, cell cycle, and cell survival, the mechanism, and function of the non-canonical activation of STAT3 by S727 phosphorylation in these processes are less well understood [66]. In agreement with the notion that the STAT3-mediated biological processes require energy, STAT3 has been shown to stimulate mitochondrial OXPHOS and the activities of electron transport chain (ETC) complex [67–70]. However, the mechanism by which STAT3 stimulates the energy production mechanism is still not clear. While some studies suggest that a small portion of STAT3 localizes to the mitochondria and promotes the ETC complex activity directly [67,69], others suggest that STAT3 does not go into the mitochondria but only closely associates with mitochondria [71]. Interestingly, here we found that STAT3 signaling is required to stimulate citrate level upon VACV infection, suggesting STAT3 signaling may indirectly promote OXPHOS and ETC through elevating the TCA cycle. Interestingly, a recent study suggests that STAT3 transcriptionally induces the citrate synthase and, hence, citrate level to regulate lymphocyte growth [18]. However, we could not observe the citrate synthase and its activity upregulation during VACV infection (not shown). More mechanistic studies are required to understand the link between STAT3 signaling and the TCA cycle activation. It is of particular interest that VACV infection selectively stimulates non-canonical STAT3 phosphorylation at the S727, but not the canonical site at Y705, although both are required for citrate level elevation. As VGF is a homolog of cellular growth factor, our result that VGF selectively stimulates EGFR-MAPK-STAT3 (S727) provides new molecular tools to understand the functions of different growth factors with diverse roles in many physiologically relevant conditions, notably, a valuable model to understand different functions and activating mechanisms of the canonical and non-canonical STAT3 signaling. Note that our results do not exclude the EGFR-MAPK-STAT3 signaling affects VACV replication other than reprogramming the TCA cycle and related metabolism. Also, STAT3 pathway is upregulated in several other viral infections [72,73,73–80]. It would be interesting to elucidate how different viruses exploit different axis of the STAT3 signaling to affect viral infections.

VGF-deleted VACV preferentially replicate in cancer cells in mice [81]. Cancer cells usually have higher and dysregulated metabolism to support cell proliferation and growth [82]. It has been noted by other studies that non-canonical activation of STAT3 at S727 is related to certain types of cancers [70,83–85]. Because most cells in animals are in resting state, in which the replication of VACV with VGF deletion is lower than in proliferating cells [34], our finding provides a metabolic mechanism of VGF-deleted VACV’s cancer cell tropism in animals.

Our results that STAT3 inhibition reduces VACV replication is somehow discrepant to a previous report that inhibition of STAT3 enhanced the replication of ACAM2000, a VACV strain currently used as a vaccine in mice, and keratinocytes [86]. We have independently confirmed the suppression effects on VACV replication using multiple inhibitors and multiple cell types [57]. We do not fully understand the discrepancy, although it could possibly be explained by different cell types or virus strains used in these studies.

Overall, we found that VACV infection elevates host cell metabolic activities, including the TCA cycle that could be achieved in a glutamine-independent manner. We identified VACV VGF as an essential viral factor that elevates the level of a central molecule of metabolism, citrate. Non-canonical STAT3 signaling is activated upon VACV infection through the VGF-EGFR-MAPK signaling axis to stimulate citrate upregulation. Our study revealed a global metabolic reprogramming effect on host cells by VACV infection and identified the cellular and viral mechanisms underlying it. The results have a broad impact on understanding poxvirus replication and prevention and understanding growth factors-induced metabolism.

## Materials and methods

### Cells and viruses

Human Foreskin Fibroblasts (HFFs) were a kind gift from Dr. Nicholas Wallace at Kansas State University and were maintained in Dulbecco’s minimal essential medium (DMEM; Fisher Scientific) supplemented with 10% fetal bovine serum (FBS; Peak Serum), 2 mM glutamine (VWR), 100 U/ml of penicillin, and 100 μg/ml streptomycin (VWR). BS-C-1 cells (ATCC CCL-26) were cultured in Eagle’s minimal essential medium (EMEM; Fisher Scientific) with supplements as described above for other cells. All cells were grown in a humidified incubator at 37°C with 5% CO_2_. VACV Western Reserve (WR) strain (ATCC VR-1354) was used in this study. Amplification, purification, and titration of VACV were performed using methods described previously [87]. Unless otherwise stated, infection of cells was performed with the indicated multiplicity of infection (MOI) of indicated viruses in special DMEM (Fisher Scientific) without glucose, L-glutamine, sodium pyruvate, and phenol red. This medium was supplemented with 2% dialyzed FBS, 1 g/L glucose (Fisher Scientific), and 2 mM glutamine. Where indicated, only glucose or glucose plus 2 mM L-asparagine was used instead of glucose plus glutamine.

### Generation of VGF (C11R) deletion and revertant VACV

VGF-deleted VACV was generated by homologous recombination by replacing the VGF-encoding C11R gene with a green fluorescent protein (GFP) gene. The GFP coding sequence following a P11 promoter flanked by 500-bp homologous sequences upstream and downstream of the C11R gene was generated by overlapping PCR and transfected into VACV-infected HeLa cells. Recombinant viruses expressing GFP were harvested from HeLa cells (ATCC CCL-2) and plaque purified in BS-C-1 cells. Recombinant VACV vΔVGF with the deletion of two copies of C11R at both ends of the virus genome was verified by PCR. The C11R revertant recombinant VACV vΔVGF_Rev was generated with a similar method by inserting a DNA fragment containing one copy of the C11R gene under the C11 promoter followed by the dsRED coding sequence under a P11 promoter into the space between the VACWR146 and VACWR147 loci in the central region of the VACV genome.

### Chemicals and antibodies

The chemical inhibitors stattic, afatinib, and PD0325901, 3-Bromopyruvate, PFK-15, and Etomoxir were purchased from Selleck chemicals and used at indicated concentrations. Cytosine-1-β-D-arabinofuranoside (AraC) and cycloheximide were purchased from Sigma-Aldrich. Ruxolitinib was purchased from VWR. CPI-613 was purchased from Biovision Inc.

Antibodies against phospho-STAT3 (S727), phospho-STAT3 (Y705), and total STAT3 were purchased from Cell Signaling Technology. Anti-glyceraldehyde-3-phosphate dehydrogenase (anti-GAPDH) antibody was purchased from Santa Cruz Biotechnology. Antibodies raised against VACV E3 protein were kind gift from Dr. Yan Xiang (UTHSA) [88]. Antibodies against VACV L2 protein were kindly provided by Dr. Bernard Moss (NIAID). A commercially synthesized recombinant VGF peptide corresponding to the cleaved fragment of VACV VGF [25] was purchased from GenScript.

### Cell viability assays

Cell viability assay was performed using the trypan blue exclusion assay as described elsewhere [89]. The cells were grown in a 12-well plate for indicated treatments were harvested with 300 μl of trypsin and resuspended with 500 μl of DMEM by pipetting. An equal volume (20 μl) of the cell suspension was gently mixed with 4% trypan blue (VWR). The number of live and dead cells in each condition was counted using a hemocytometer.

### Measurement of citrate, oxaloacetate (OAA), Acetyl-CoA, and ATP

The citrate measurement was carried out using EnzyChrom Citrate Assay Kit (BioAssay Systems) according to the manufacturer’s instructions. 4x10^6^ HFFs were collected in 100 μl of ice-cold PBS. The cells were homogenized by sonication, and the cell lysis was verified by observation under a microscope. The lysed cell suspension was centrifuged at 19,000 xg at 4°C for 5 min. Twenty μl of the clear supernatant was mixed with 80 μl of fresh working reagent and in a 96-well black clear bottom plate (Corning) and incubated protected from light at room temperature for 15 minutes. Fluorescence reading at λex/em = 535/595 nm was measured, and the level of citrate in the sample was calculated using a standard curve generated alongside each experiment.

For the measurement of OAA, we followed the protocols outlined in the Oxaloacetate Assay Kit (Sigma-Aldrich). Briefly, 4x10^6^ HFFs were collected and homogenized in the assay buffer. The sample was centrifuged at 15,000 x g for 10 min at 4°C. After mixing 50 μl of the fresh working reagent with 50 μl of the deproteinized sample, the mixture was incubated at room temperature for 30 min. Finally, Fluorescence reading of samples, standards, and controls was measured at λex/em = 535/595 nm, and the level of OAA in the sample was calculated.

The level of Acetyl-CoA was measured using the PicoProbe Acetyl CoA Assay Kit (Abcam) according to the manufacturer’s instructions. Briefly, 4x10^6^ HFFs were collected and homogenized in the assay buffer in ice. The cells were lysed by sonication, and the sample was centrifuged at 10,000 xg for 10 min at 4°C. The supernatant was collected and then deproteinized with a perchloric acid method. Then, ten μl of the deproteinized sample was added to each well, and the final volume was brought up to 50 μl with assay buffer. The coenzyme A present was quenched by adding a quencher for 5 minutes, and eventually, it was removed with quencher remover. Finally, 50 μl of fresh reaction mixture was added to the above samples, and the mixture was incubated at 37°C for half an hour. Fluorescence reading of samples, standards, and controls was measured at λex/em = 535/595 nm to calculate the level of Acetyl-CoA in the sample.

The levels of ATP were measured using an ATP Detection Assay Kit–Luminescence (Cayman Chemical Company). Briefly, after desired treatment, 4x10^5^ HFFs were washed with ice-cold 1x PBS and homogenized in 500 μL prechilled 1x ATP detection sample buffer. After mixing 100 μl of the fresh reaction mixture (containing D-Luciferin and ATP detection luciferase) with 10 μl of the sample, standards, or blank, the mixture was incubated at room temperature for 15 min protected from light. Finally, the luminescence was measured using a luminometer and the ATP levels in the sample was calculated using a standard curve generated alongside each experiment.

### Global metabolic profiling

Metabolic profiling was carried out with Metabolon, as described previously [38]. Briefly, four biological replicates of each treatment were used for each treatment. HFFs were grown in T-175 flasks. Once the cells reached about 95% confluence, they were washed twice with 1x PBS at 37°C and infected with VACV at an MOI of 3 and cultured in different media. At 8 and 16 hpi, the cells were harvested by scraping, and the pellets were washed twice in ice-cold 1x PBS. The pellet was then dissolved in the extraction solvent (methanol) and was stored at −80°C until shipment to Metabolon (Durham, North Carolina). Proprietary analytical procedures were carried to ensure high-quality data after minimizing the system artifacts, misassignments, and background noise among the samples. Following normalization to the protein concentration, log transformation, and imputation of missing values, with the minimum observed value for each compound, ANOVA contrasts were used to identify biochemicals that differed significantly between experimental groups.

### Western blotting analysis

Western blot was performed as described previously [90]. Briefly, the cells were lysed in NP-40 cell lysis buffer after the required treatment, reduced with 100 mM dithiothreitol (DTT), and denatured by sodium dodecyl sulfate-polyacrylamide gel electrophoresis (SDS–PAGE) loading buffer. After boiling at 99°C for 5 min, the samples were loaded on the SDS–PAGE, followed by transferring to a polyvinylidene difluoride membrane. The membrane was blocked in 5% bovine serum albumin (BSA; VWR) blocking buffer in TBST buffer for 1 h at room temperature and incubated with the primary antibody in the same BSA blocking buffer for overnight at 4°C. After 3x washes of 10 minutes each with TBST, the membrane was incubated with horseradish peroxidase-conjugated secondary antibody for 1 h at room temperature. The membranes were developed with Thermo Scientific SuperSignal West Femto Maximum Sensitivity Substrate. Antibodies were stripped from the membrane by Restore (Thermo Fisher Scientific, Waltham, MA, United States) for Western blotting analysis using another antibody.

### *Gaussia* luciferase assay

The *Gaussia* luciferase activity assay was performed as previously described [38]. Briefly, cells were infected with a recombinant VACV encoding *Gaussia* luciferase under the VGF (C11R) viral early promoter (vEGluc) for indicated time. The cell culture media was used to measure the *Gaussia* luciferase activities assay using Pierce Gaussia luciferase flash assay kit (Thermo Scientific) and a luminometer.

### Plaque assay and plaque size determination

BS-C-1 cell monolayers were infected with indicated viruses. One hour post infection, the media was changed to EMEM containing supplements as described above plus 0.5% methylcellulose (Fisher Scientific). The viruses were allowed to grow and form plaque for 48 hrs. The growth medium was discarded, and the cells were treated with 0.1% (w/v) crystal violet (Fisher Scientific) in 20% ethanol for 10 minutes. The image of plate containing plaques was taken and the plaque diameters were measured using the ImageJ software (version 1.51w) [91]. The diameter of 50 plaques were measured per condition and the data was analyzed in RStudio (version 1.2.5033) [92].

### Quantitative reverse transcription PCR (qRT-PCR)

Total RNA was extracted from cells using TRIzol reagent (Ambion), and then it was purified using the Invitrogen PureLink RNA mini kit (Thermo Fisher Scientific). 500 ng RNA was used as a template to reverse transcribe into cDNA using random hexamer primers and SuperScript III first-strand synthesis kit (Invitrogen). CFX96 Real-Time PCR Detection System (Bio-Rad) with All-in-One 2X quantitative PCR (qPCR) mix (GeneCopoeia) and primers specific for indicated genes was used to detect the relative levels of indicated mRNAs in the sample using following settings: Initial denaturation at 95°C for 3 min, followed by 39 cycles of denaturation at 95°C for 10 s, annealing and reading fluorescence at 52°C for 30 s, and extension at 72°C for 30 s. 18sRNA was used as an internal control for normalization.

### RNA interference

The indicated specific siRNAs and negative control siRNAs were purchased from Qiagen. The siRNAs were transfected at a concentration of 5 nM in Lipofectamine RNAiMAX transfection reagent (Fisher Scientific) following the manufacturer’s instructions. The efficiency of knockdown was measured by Western blotting analysis.

### Statistical analyses

Data presented indicate a mean of at least three biological replicates, unless otherwise stated. For the global metabolic profiling, four biological repeats were used for each condition, and the data was analyzed and visualized in RStudio (version 1.2.5033) [92] and MetaboAnalyst software [93]. Error bars indicate the standard deviation of the experimental replicates. A two-tailed paired *t*-test was performed to evaluate any significant difference between the two means. We used the following convention for symbols to indicate statistical significance: ns, *P* > 0.05; *, *P* ≤ 0.05; **, *P* ≤ 0.01; ***, *P* ≤ 0.001; ****, *P* ≤ 0.0001.

## Supporting information

S1 Fig (A) Heatmap of VACV-induced alteration of metabolism in HFFs in medium with glucose plus asparagine. (B) Heatmap of VACV-induced alteration of metabolism in medium with glucose only. Color keys indicate the levels of different metabolites; blue: lowest, red: highest.
(TIF)

S2 Fig VACV infection increases the levels of most of the TCA cycle intermediates in the absence of glutamine in the medium. HFFs infected with VACV at an MOI of 3 of in medium with glucose only (Glc) or glucose+asparagine (Glc+N). The levels of TCA cycle intermediates at 16 hpi were measured by performing metabolic profiling. ns, P > 0.05; *, P ≤ 0.05; **, P ≤ 0.01; ***, P ≤ 0.001; ****, P ≤ 0.0001.
(TIF)

S3 Fig VACV infection decreases the level of acetyl-CoA. HFFs infected with VACV at an MOI of 3 in medium with glucose only (Glc) or glucose + asparagine (Glc+N). The level of acetyl CoA at 16 hpi was measured by performing metabolic profiling. Error bars represent the standard deviation of four biological replicates. *, P ≤ 0.05; ***, P ≤ 0.001.
(TIF)

S4 Fig **(A)** Outline of glycolysis pathway. Glucose after a series of reactions is converted into pyruvate, which can then either be converted to lactate under anaerobic conditions or to acetyl coenzyme A under aerobic conditions. **(B)** The glycolysis intermediates are either unaffected or reduced during VACV infection. The levels of glycolysis intermediates in HFFs infected with MOI-3 of WT-VACV in media with glucose (Glc) or glucose plus asparagine (Glc+N) at 16 hpi as determined by global metabolic profiling in Fig 1A. ns, P > 0.05; *, P ≤ 0.05; **, P ≤ 0.01; ***, P ≤ 0.001.
(TIF)

S5 Fig VACV infection does not significantly affect the level of lactate. The level of lactate in HFFs infected with MOI = 3 of WT-VACV in media with glucose (Glc) or glucose plus asparagine (Glc+N) at 8 hpi was determined by global metabolic profiling in Fig 1A. Error bars represent the standard deviation of four biological replicates. ns, P > 0.05.
(TIF)

S6 Fig **(A)** VGF deletion reduces VACV replication in HFFs. HFFs infected with indicated viruses at MOI of 0.001 in medium with glucose plus glutamine with 0.001% dialyzed FBS. Virus titers measured at 72 hpi using a plaque assay. **(B)** VGF deletion decreases plaque size. The virus samples acquired from S6 Fig (A) were used to infect a confluent monolayer of BS-C-1 cells for 48 h. The diameters of 50 plaques from each treatment were measured and analyzed as described in the Materials and Methods section. Error bars represent the standard deviation of at least three biological replicates in (A) and 50 plaques in (B). ns, P > 0.05; *, P ≤ 0.05; ****, P ≤ 0.0001.
(TIF)

S7 Fig A synthetic VGF peptide alone did not enhance the levels of citrate in HFFs. HFFs were either mock-infected, infected with indicated viruses at an MOI of 5 or treated with 2500 ng/mL of a synthetic VGF peptide. After 4 h of treatment, citrate levels in the samples were measured by a citrate assay kit. Error bars represent the standard deviation of at least three biological replicates. *, P ≤ 0.05; **, P ≤ 0.01.
(TIF)

S8 Fig **(A)** HFFs were grown in the presence or absence of 3 μM afatinib for 24 h. Cell viability was measured using a trypan blue exclusion assay. **(B)** Inhibition of the EGFR pathway reduces VACV titers. HFFs were infected with VACV at an MOI of 2 in the presence or absence of 3 μM afatinib for 24 h. Virus titers were measured using a plaque assay. Error bars represent the standard deviation of at least three biological replicates. ns, P > 0.05; **, P ≤ 0.01.
(TIF)

S9 Fig **(A)** Inhibition of the MAPK pathway suppresses VACV replication. HFFs were infected with VACV at an MOI of 2 in the presence or absence of 50 μM PD0325901 for 24 h. A plaque assay was performed to measure virus titers. **(B)** Inhibition of the MAPK pathway does not decrease HFF viability. HFFs were grown in for 24 h in the presence or absence of 50 μM PD0325901. Cell viability was determined using a trypan blue exclusion assay. Error bars represent the standard deviation of at least three biological replicates. ns, P > 0.05; ***, P ≤ 0.001.
(TIF)

S10 Fig (A) Inhibition of the STAT3 pathway suppresses VACV replication. HFFs were infected with WT VACV at an MOI of 2 in the presence or absence of 3 μM stattic for 24 h. VACV titers were measured using a plaque assay. (**B)** HFFs were grown in the presence or absence of 3 μM stattic for 24 h. Cell viability was determined using a trypan blue exclusion assay. (**C**) STAT3 knockdown does not affect HFF viability. HFFs were transfected with indicated siRNAs for 72 h, and a trypan blue exclusion assay was performed to determine the cell viability. Error bars represent the standard deviation of at least three biological replicates. ns, P > 0.05; **, P ≤ 0.01.
(TIF)

S11 Fig Inhibition of the JAK1/2 pathway does not alter HFF viability. HFFs were grown in the presence or absence of 50 μM ruxolitinib for 24 h. Cell viability was determined by a trypan blue exclusion assay using a hemocytometer. All experiments were performed in media with glucose plus glutamine. Error bars represent the standard deviation of at least three biological replicates. ns, P > 0.05.
(TIF)

S1 File The number of metabolites significantly different upon VACV infection in medium with glucose or glucose plus asparagine. The numbers approaching a significant difference are also shown in the lower two rows. The red upward arrows indicate increase and the green downwards arrows indicate decrease in indicated conditions.
(XLSX)

S2 File Biochemicals profiled in this study. Red and green shaded cells indicate p≤0.05 (red indicates that the mean values are significantly higher for that comparison; green values significantly lower). Light red and light green shaded cells indicate 0.05<p<0.10 (light red indicates that the mean values trend higher for that comparison; light green values trend lower).
(XLSX)
