# Supplementary figures and images for: Viral growth factor- and STAT3 signaling-dependent elevation of the TCA cycle intermediate levels during vaccinia virus infection
Source: PLoS Pathog. 2021 Feb 2;17(2):e1009303. doi: 10.1371/journal.ppat.1009303 (PMC7880457; doi:10.1371/journal.ppat.1009303)

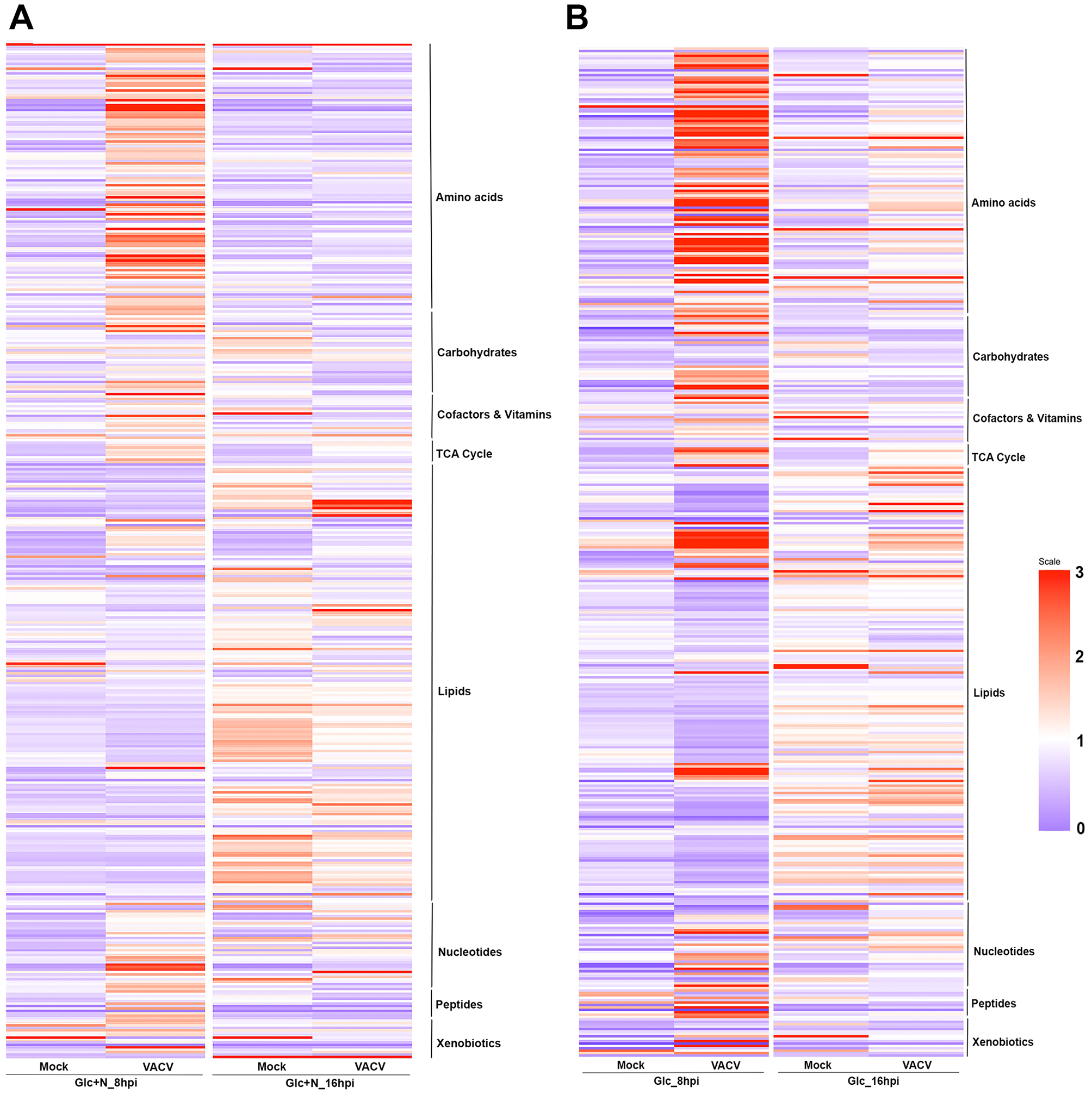

Supplement: S1 Fig — (A) Heatmap of VACV-induced alteration of metabolism in HFFs in medium with glucose plus asparagine. (B) Heatmap of VACV-induced alteration of metabolism in medium with glucose only. Color keys indicate the levels of different metabolites; blue: lowest, red: highest. (TIF) [file ppat.1009303.s001.tif]

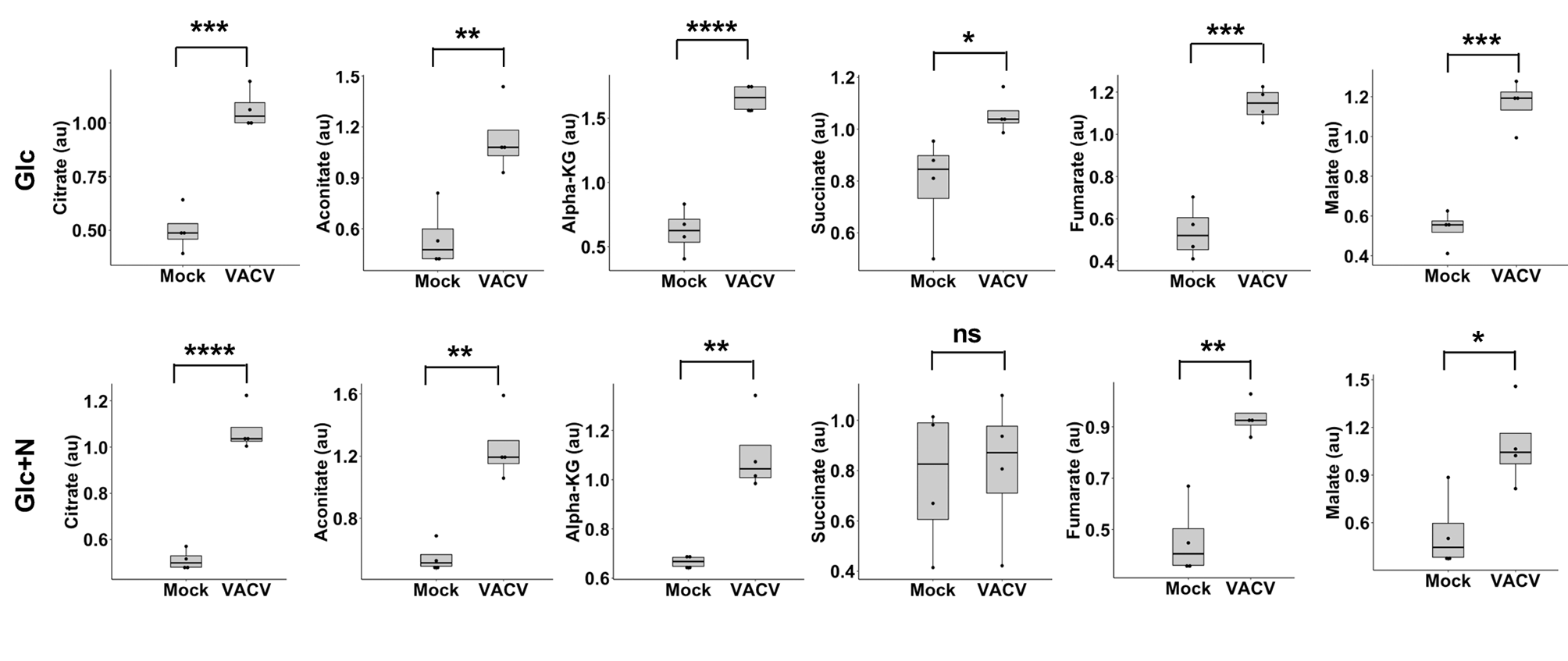

Supplement: S2 Fig — HFFs infected with VACV at an MOI of 3 of in medium with glucose only (Glc) or glucose+asparagine (Glc+N). The levels of TCA cycle intermediates at 16 hpi were measured by performing metabolic profiling. ns, P > 0.05; *, P ≤ 0.05; **, P ≤ 0.01; ***, P ≤ 0.001; ****, P ≤ 0.0001. (TIF) [file ppat.1009303.s002.tif]

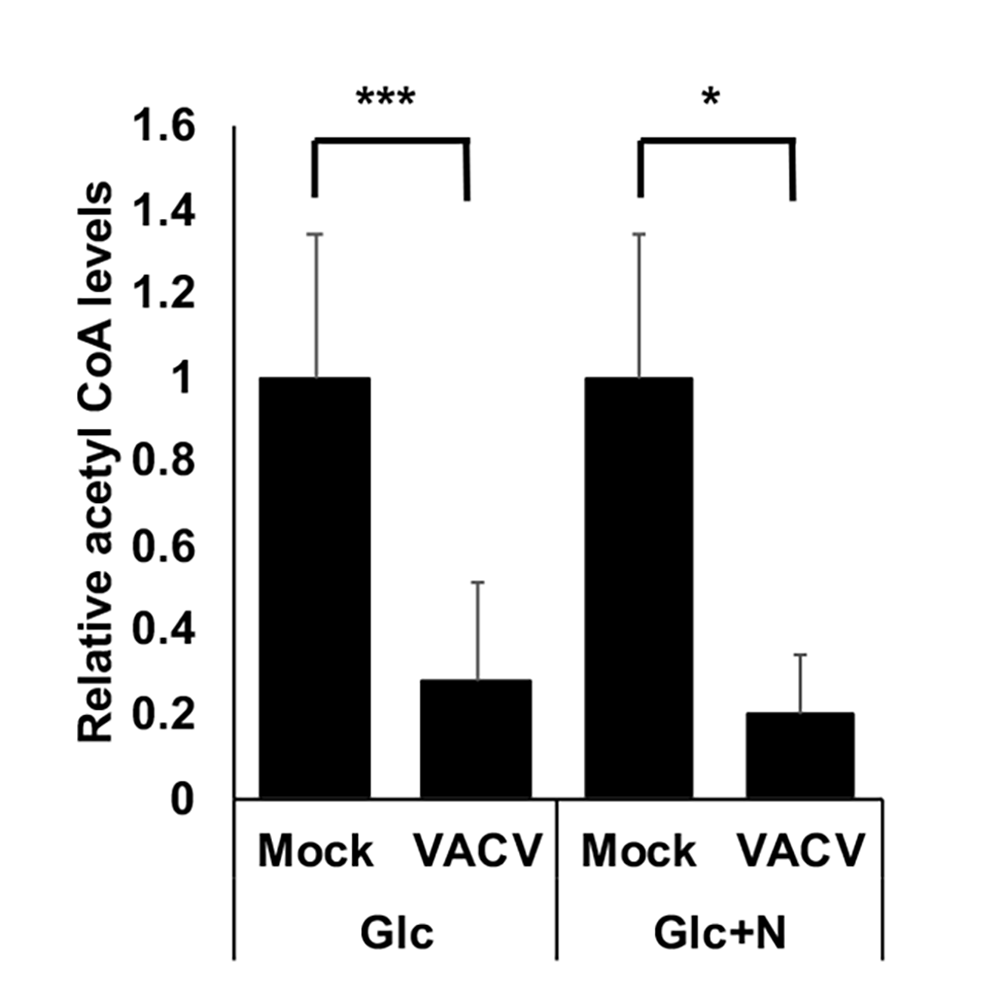

Supplement: S3 Fig — HFFs infected with VACV at an MOI of 3 in medium with glucose only (Glc) or glucose + asparagine (Glc+N). The level of acetyl CoA at 16 hpi was measured by performing metabolic profiling. Error bars represent the standard deviation of four biological replicates. *, P ≤ 0.05; ***, P ≤ 0.001. (TIF) [file ppat.1009303.s003.tif]

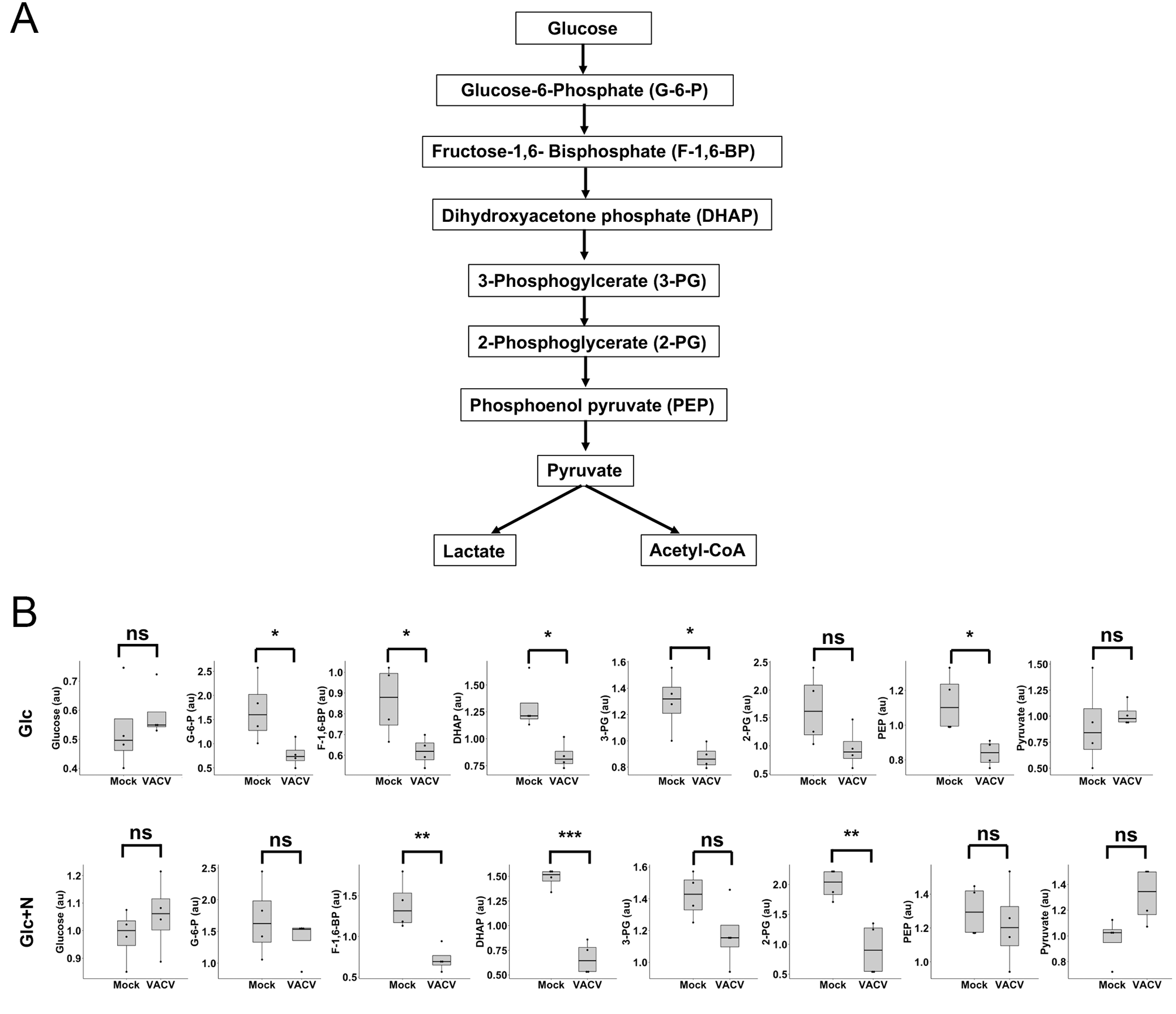

Supplement: S4 Fig — (A) Outline of glycolysis pathway. Glucose after a series of reactions is converted into pyruvate, which can then either be converted to lactate under anaerobic conditions or to acetyl coenzyme A under aerobic conditions. (B) The glycolysis intermediates are either unaffected or reduced during VACV infection. The levels of glycolysis intermediates in HFFs infected with MOI-3 of WT-VACV in media with glucose (Glc) or glucose plus asparagine (Glc+N) at 16 hpi as determined by global metabolic profiling in Fig 1A. ns, P > 0.05; *, P ≤ 0.05; **, P ≤ 0.01; ***, P ≤ 0.001. (TIF) [file ppat.1009303.s004.tif]

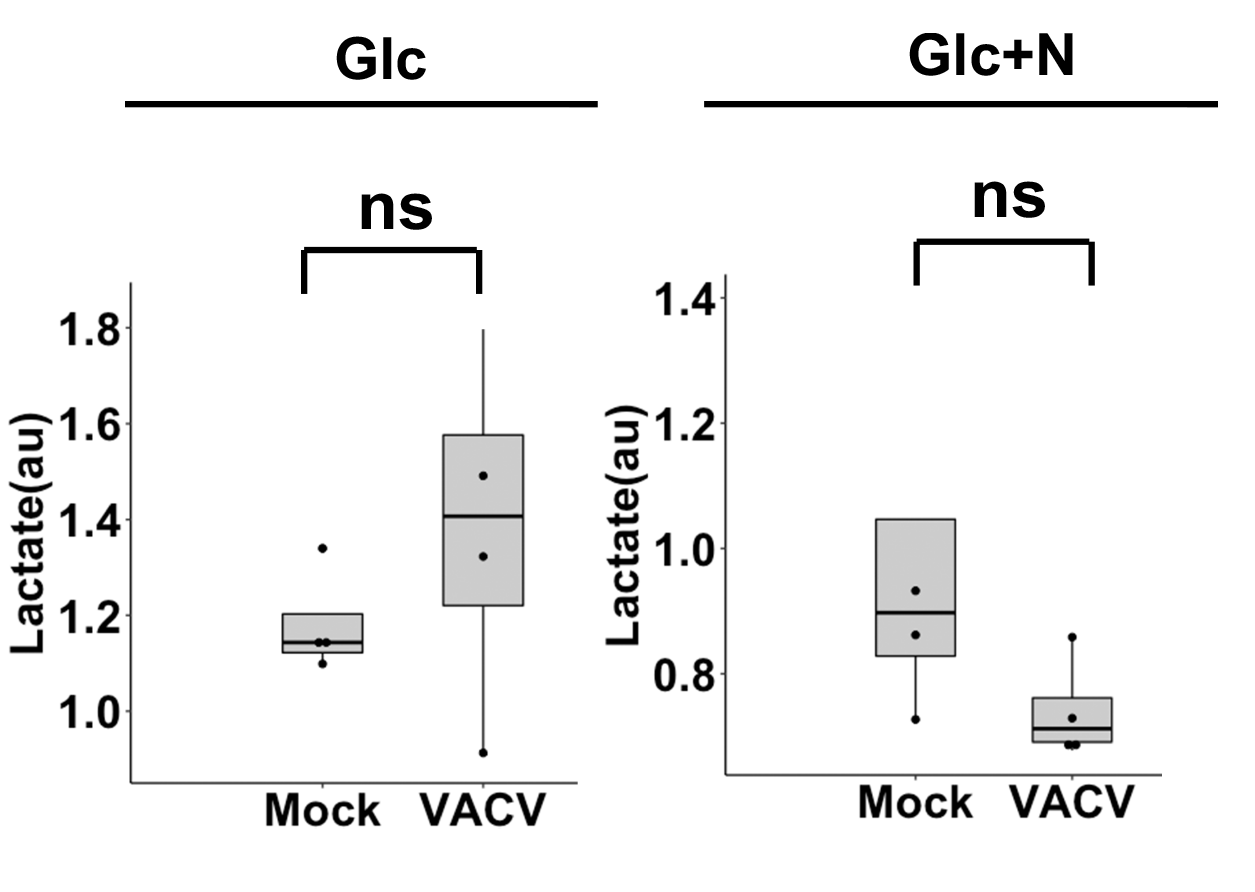

Supplement: S5 Fig — The level of lactate in HFFs infected with MOI = 3 of WT-VACV in media with glucose (Glc) or glucose plus asparagine (Glc+N) at 8 hpi was determined by global metabolic profiling in Fig 1A. Error bars represent the standard deviation of four biological replicates. ns, P > 0.05. (TIF) [file ppat.1009303.s005.tif]

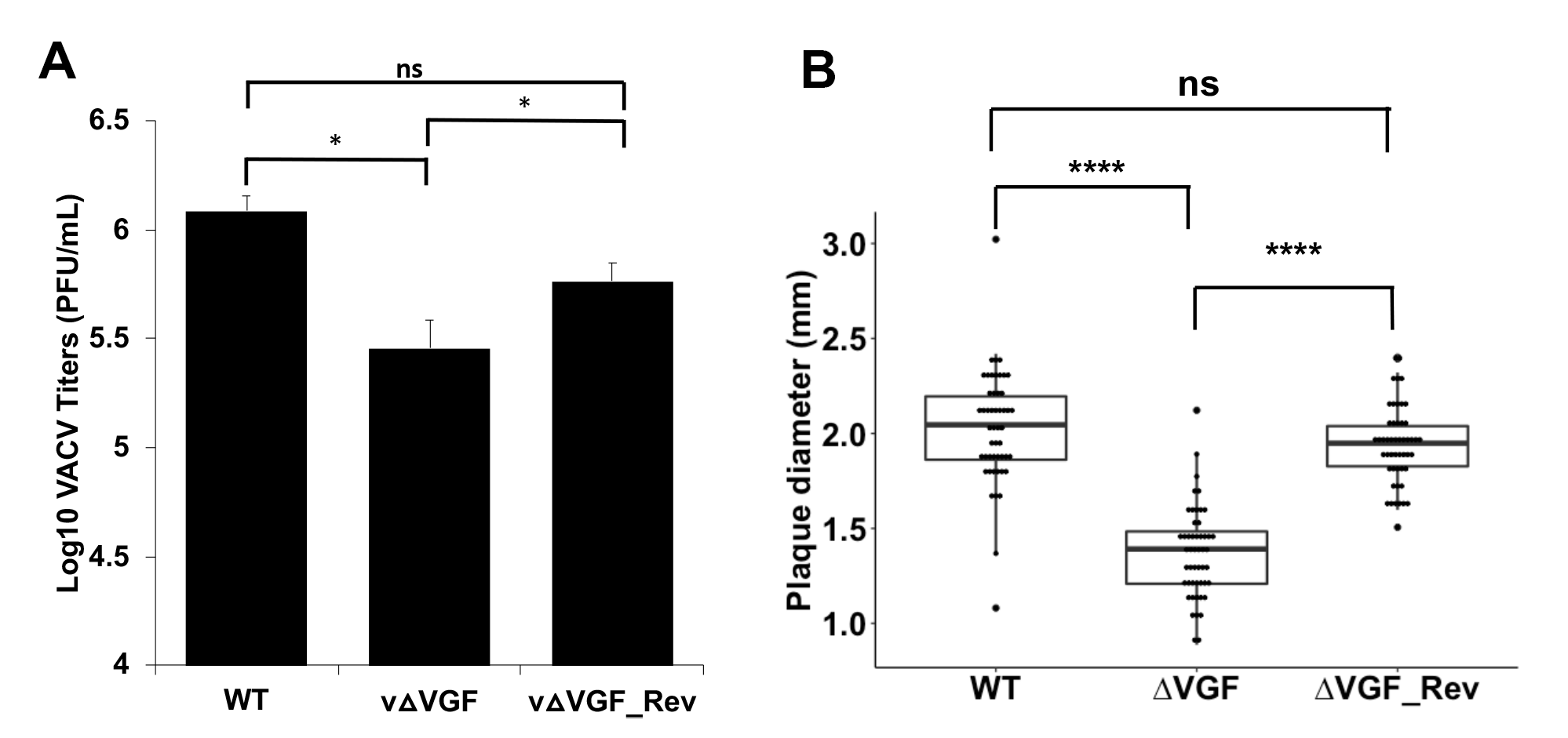

Supplement: S6 Fig — (A) VGF deletion reduces VACV replication in HFFs. HFFs infected with indicated viruses at MOI of 0.001 in medium with glucose plus glutamine with 0.001% dialyzed FBS. Virus titers measured at 72 hpi using a plaque assay. (B) VGF deletion decreases plaque size. The virus samples acquired from S6 Fig (A) were used to infect a confluent monolayer of BS-C-1 cells for 48 h. The diameters of 50 plaques from each treatment were measured and analyzed as described in the Materials and Methods section. Error bars represent the standard deviation of at least three biological replicates in (A) and 50 plaques in (B). ns, P > 0.05; *, P ≤ 0.05; ****, P ≤ 0.0001. (TIF) [file ppat.1009303.s006.tif]

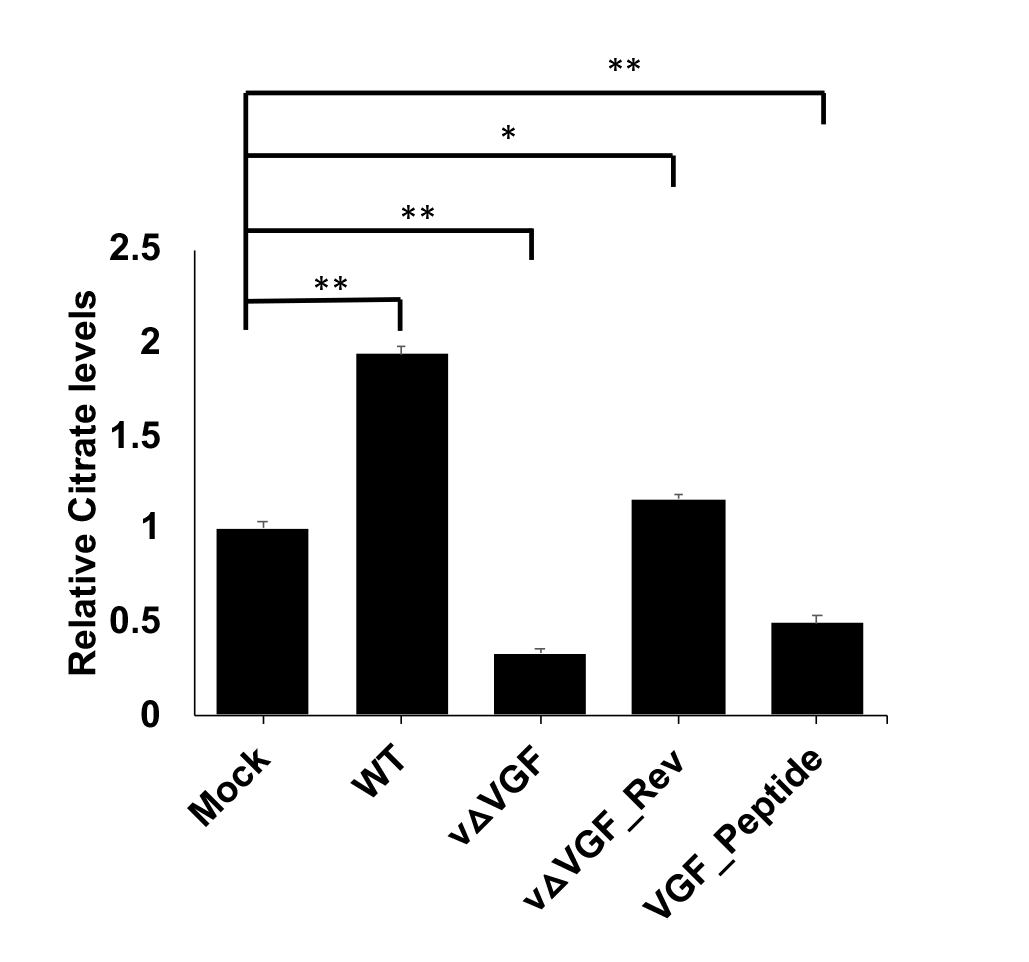

Supplement: S7 Fig — HFFs were either mock-infected, infected with indicated viruses at an MOI of 5 or treated with 2500 ng/mL of a synthetic VGF peptide. After 4 h of treatment, citrate levels in the samples were measured by a citrate assay kit. Error bars represent the standard deviation of at least three biological replicates. *, P ≤ 0.05; **, P ≤ 0.01. (TIF) [file ppat.1009303.s007.tif]

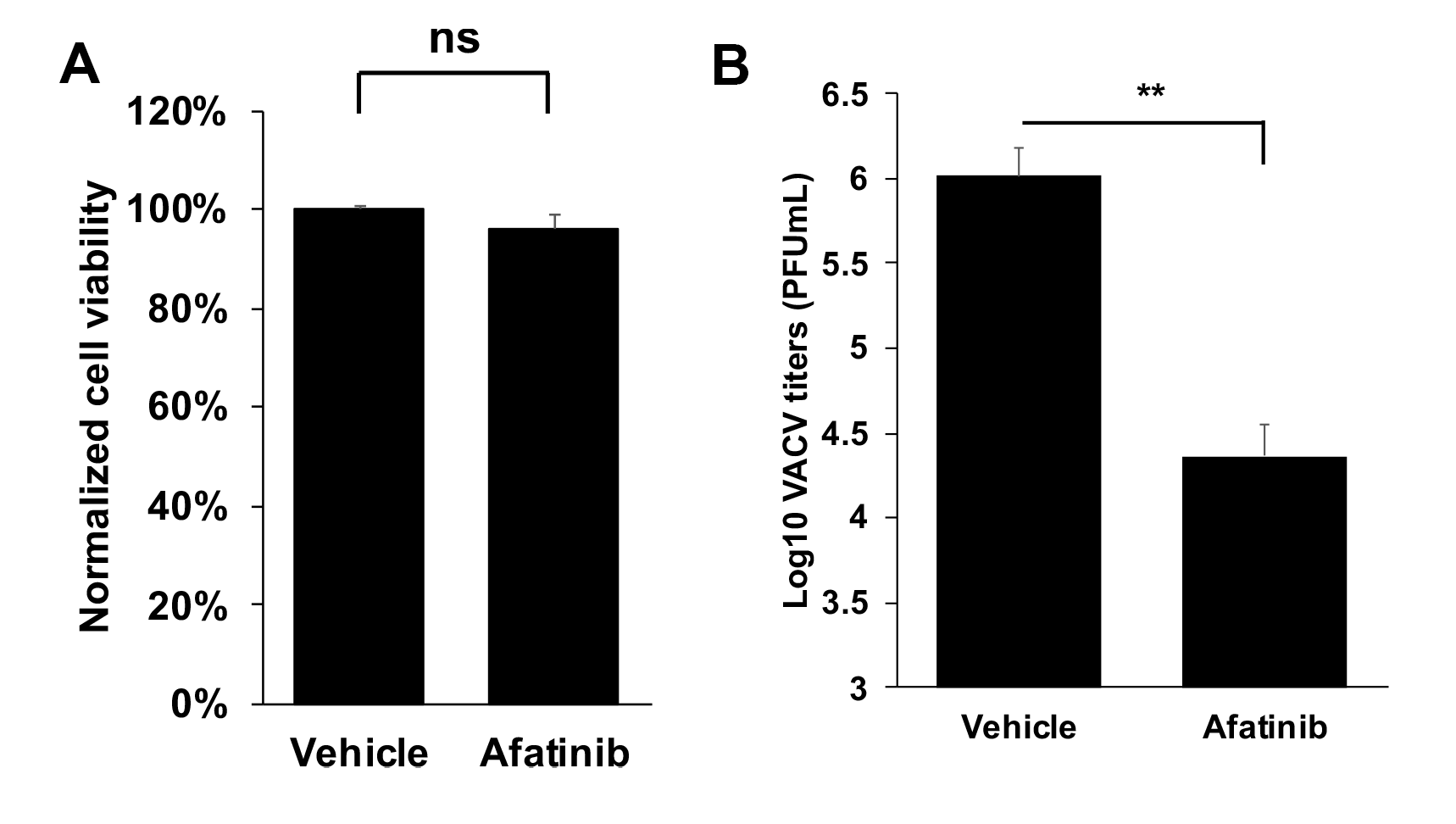

Supplement: S8 Fig — (A) HFFs were grown in the presence or absence of 3 μM afatinib for 24 h. Cell viability was measured using a trypan blue exclusion assay. (B) Inhibition of the EGFR pathway reduces VACV titers. HFFs were infected with VACV at an MOI of 2 in the presence or absence of 3 μM afatinib for 24 h. Virus titers were measured using a plaque assay. Error bars represent the standard deviation of at least three biological replicates. ns, P > 0.05; **, P ≤ 0.01. (TIF) [file ppat.1009303.s008.tif]

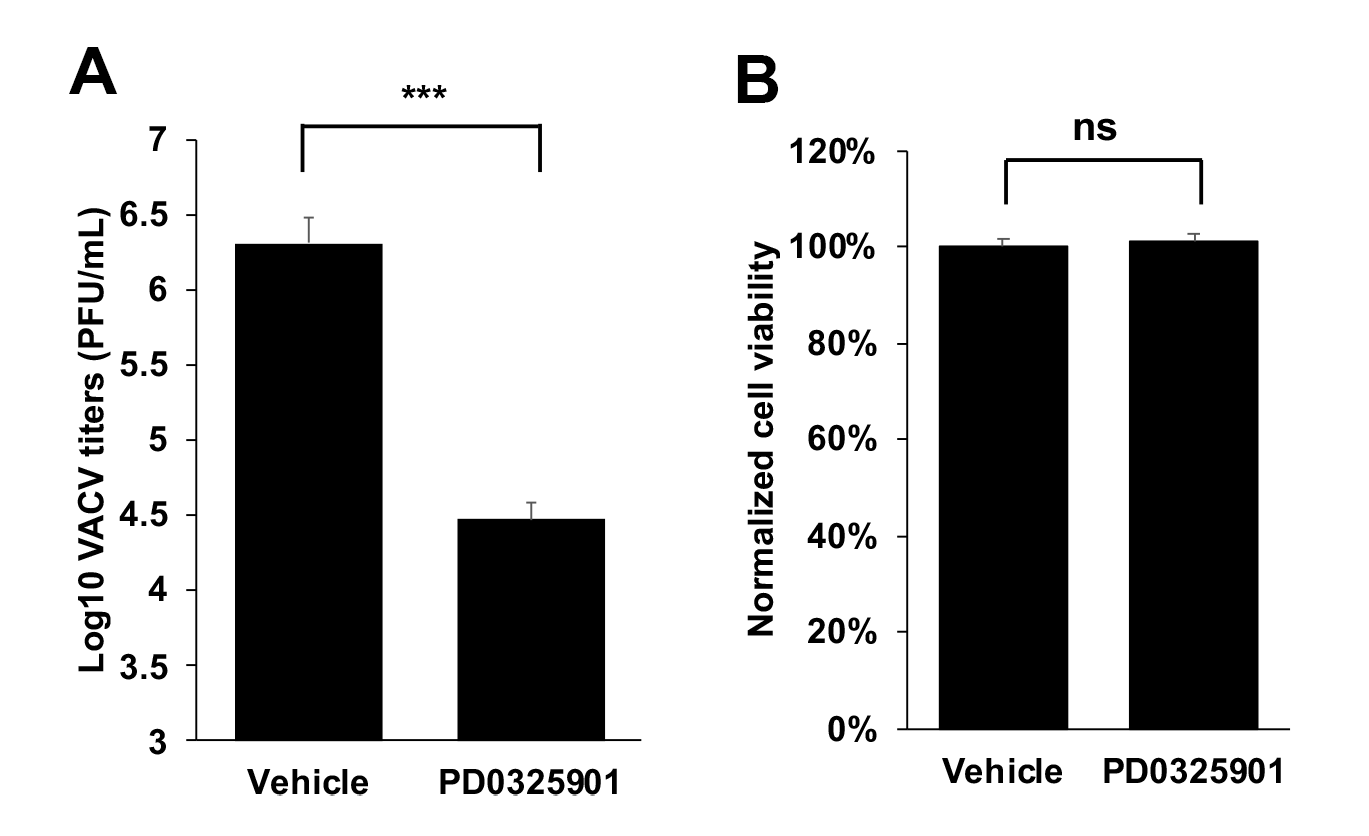

Supplement: S9 Fig — (A) Inhibition of the MAPK pathway suppresses VACV replication. HFFs were infected with VACV at an MOI of 2 in the presence or absence of 50 μM PD0325901 for 24 h. A plaque assay was performed to measure virus titers. (B) Inhibition of the MAPK pathway does not decrease HFF viability. HFFs were grown in for 24 h in the presence or absence of 50 μM PD0325901. Cell viability was determined using a trypan blue exclusion assay. Error bars represent the standard deviation of at least three biological replicates. ns, P > 0.05; ***, P ≤ 0.001. (TIF) [file ppat.1009303.s009.tif]

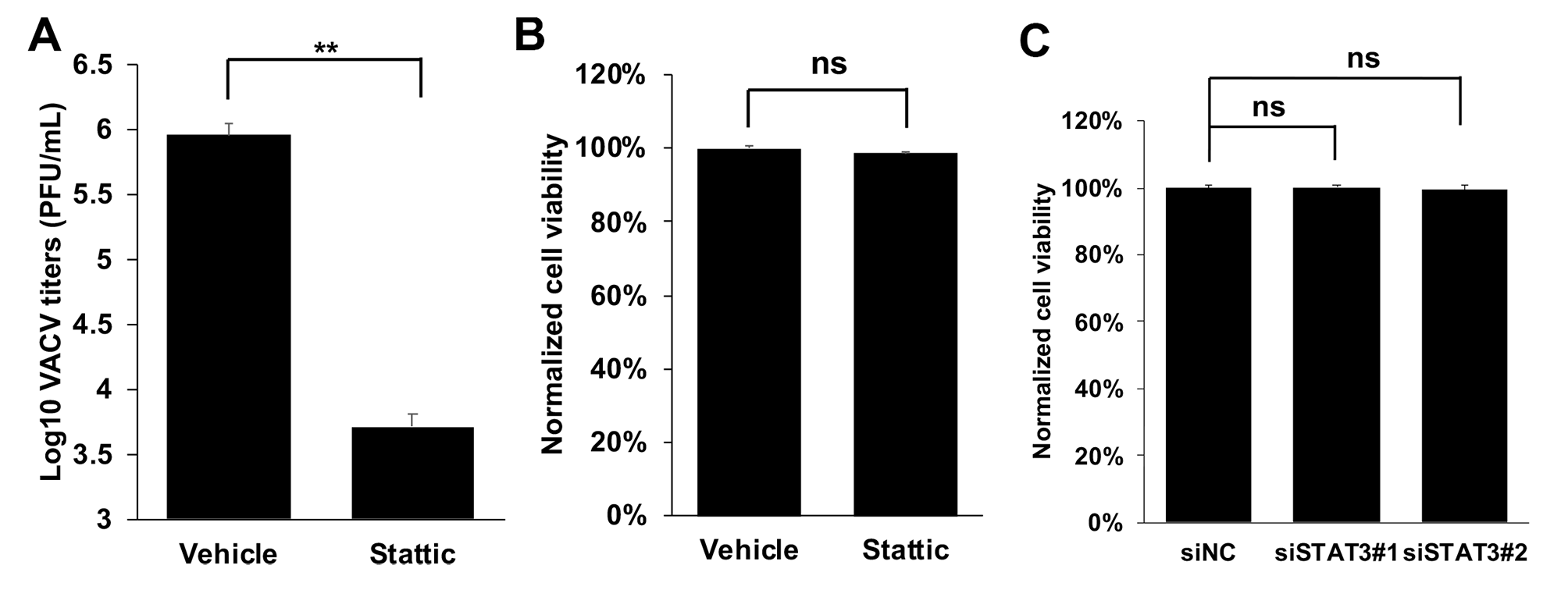

Supplement: S10 Fig — (A) Inhibition of the STAT3 pathway suppresses VACV replication. HFFs were infected with WT VACV at an MOI of 2 in the presence or absence of 3 μM stattic for 24 h. VACV titers were measured using a plaque assay. (B) HFFs were grown in the presence or absence of 3 μM stattic for 24 h. Cell viability was determined using a trypan blue exclusion assay. (C) STAT3 knockdown does not affect HFF viability. HFFs were transfected with indicated siRNAs for 72 h, and a trypan blue exclusion assay was performed to determine the cell viability. Error bars represent the standard deviation of at least three biological replicates. ns, P > 0.05; **, P ≤ 0.01. (TIF) [file ppat.1009303.s010.tif]

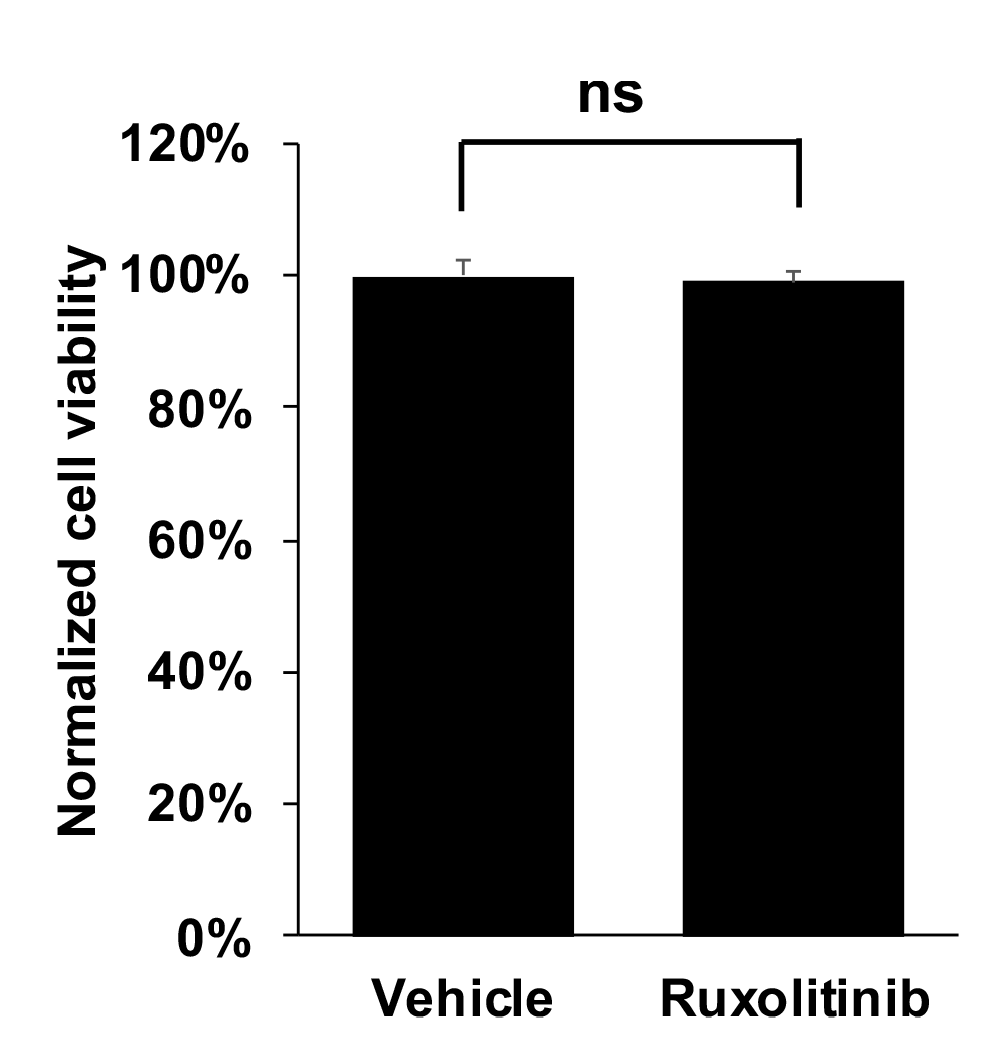

Supplement: S11 Fig — HFFs were grown in the presence or absence of 50 μM ruxolitinib for 24 h. Cell viability was determined by a trypan blue exclusion assay using a hemocytometer. All experiments were performed in media with glucose plus glutamine. Error bars represent the standard deviation of at least three biological replicates. ns, P > 0.05. (TIF) [file ppat.1009303.s011.tif]
